# Ciliogenesis defects after neurulation impact brain development and neuronal activity in larval zebrafish

**DOI:** 10.1016/j.isci.2024.110078

**Published:** 2024-05-22

**Authors:** Percival P. D’Gama, Inyoung Jeong, Andreas Moe Nygård, Anh-Tuan Trinh, Emre Yaksi, Nathalie Jurisch-Yaksi

**Affiliations:** 1 Department of Clinical and Molecular Medicine, Norwegian University of Science and Technology, Erling Skalgssons gate 1, 7030 Trondheim, Norway; 2 Kavli Institute for Systems Neuroscience and Centre for Algorithms in the Cortex, Norwegian University of Science and Technology, Olav Kyrres Gate 9, 7030 Trondheim, Norway; 3 Koç University Research Center for Translational Medicine, Koç University School of Medicine, Davutpaşa Caddesi, No:4, Topkapı 34010, Istanbul, Turkey

**Keywords:** Biological sciences, Neuroscience, Developmental neuroscience

## Abstract

Cilia are slender, hair-like structures extending from cell surfaces and playing essential roles in diverse physiological processes. Within the nervous system, primary cilia contribute to signaling and sensory perception, while motile cilia facilitate cerebrospinal fluid flow. Here, we investigated the impact of ciliary loss on neural circuit development using a zebrafish line displaying ciliogenesis defects. We found that cilia defects after neurulation affect neurogenesis and brain morphology, especially in the cerebellum, and lead to altered gene expression profiles. Using whole brain calcium imaging, we measured reduced light-evoked and spontaneous neuronal activity in all brain regions. By shedding light on the intricate role of cilia in neural circuit formation and function in the zebrafish, our work highlights their evolutionary conserved role in the brain and sets the stage for future analysis of ciliopathy models.

## Highlights

• *traf3ip1/elipsa* mutant zebrafish display ciliogenesis defects after neurulation
• Cilia defects lead to abnormal brain morphology, cell division, and gene expression
• Cerebellar malformations and reduced number of Purkinje cells upon cilia defects
• Reduced light-evoked and spontaneous neural activity in cilia mutants

## Graphical abstract

**Figure:**
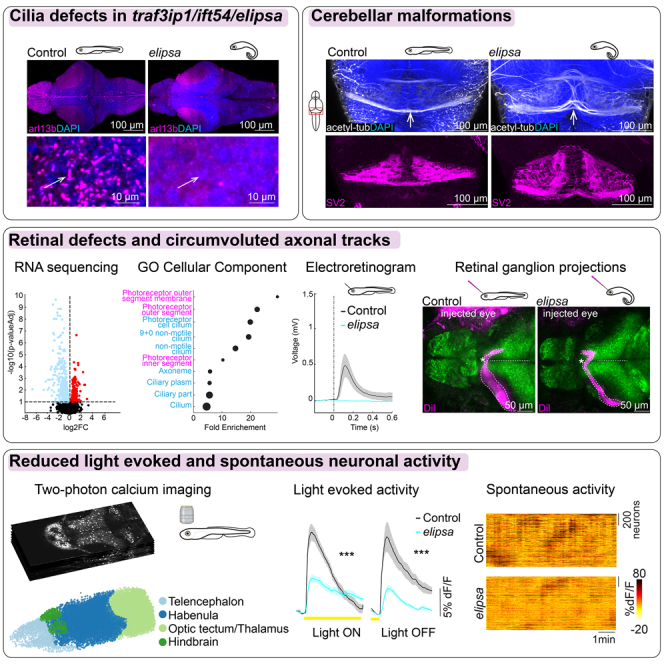

## Introduction

Cilia are slender, hair-like structures that extend from the surface of cells.^1^^,^^2^ They play a vital role in various physiological processes across species.^3^^,^^4^ In the context of the nervous system, cilia are involved in functions ranging from cellular signaling to sensory perception and movement of cerebrospinal fluid (CSF).^5^^,^^6^^,^^7^^,^^8^^,^^9^^,^^10^^,^^11^

Primary cilia are present on neuronal progenitors and have been described in many species, including human, mouse, and zebrafish.^12^^,^^13^^,^^14^ They serve as essential signaling hubs which receive and transmit cues,^15^^,^^16^ such as hedgehog signaling.^17^^,^^18^ Cilia have been associated with the control of proliferation and differentiation,^14^^,^^19^ patterning,^20^ migration,^21^^,^^22^^,^^23^ axon guidance,^10^^,^^24^ synaptogenesis,^25^ and connectivity.^26^^,^^27^

In differentiated neurons, primary cilia play a dual role. They can participate in sensing sensory modalities, such as odors^8^^,^^28^^,^^29^^,^^30^ and light,^9^^,^^31^^,^^32^ or they can actively influence neuronal physiology.^11^^,^^33^^,^^34^^,^^35^ For example, cilia were shown to regulate food uptake through the modulation of hypothalamic neurons in the paraventricular nucleus^35^ and circadian rhythm through interneuronal coupling in the suprachiasmatic nucleus.^36^ Cilia can also alter the chromatin accessibility of hippocampal neurons through axo-ciliary serotonergic synapses.^34^ Ciliary signaling usually involves G protein-coupled receptors, which localize to the primary cilium, such as olfactory receptors, somatostatin receptor type 3 (SSTR3), serotonin receptor 6 (HTR6), melanin-concentrating hormone receptor 1 (MCHR1), or dopamine receptor 1 (D1).^11^^,^^15^^,^^34^^,^^37^^,^^38^^,^^39^^,^^40^^,^^41^^,^^42^^,^^43^

In contrast to primary cilia, motile cilia propel fluids and particles across tissue surfaces.^44^^,^^45^ In the brain, cilia-driven fluid flow is important for the movement and homeostasis of CSF.^6^^,^^46^^,^^47^^,^^48^^,^^49^^,^^50^ Intriguingly, CSF flow has also been implicated in neuronal migration in mice, through the potential establishment of morphogen gradients.^49^ Cilia-mediated CSF flow has also been linked to the regulation of neuronal activity in the larval zebrafish brain.^51^

Human patients with dysfunctional cilia can present several neurodevelopmental and neurological symptoms.^10^^,^^52^^,^^53^ For instance, mutations in the genes encoding Bardet-Biedl syndrome (BBS) proteins are associated with retinal degeneration, hyperphagia, and learning disabilities.^54^^,^^55^ Another classical example is Joubert syndrome,^56^^,^^57^^,^^58^ which is a rare genetic disorder affecting the development of the cerebellum and brainstem.^59^^,^^60^ Joubert syndrome is characterized by cerebellar vermis malformation, disrupting motor coordination in addition to cognitive dysfunction and epilepsy.^56^^,^^61^ Altogether, these findings suggest that disruptions in ciliary function impact neuronal development, which may influence neuronal activity in the brain and even lead to epileptic seizures. However, it is still unclear how primary cilia loss affects the establishment of neuronal circuits in the brain and whether it leads to altered neural activity.

In this study, we leveraged the small size, transparency, and genetic amenability of zebrafish larvae to investigate the impact of ciliary loss on the development of neural circuits. To this end, we used a previously characterized cilia mutant carrying a mutation in the ciliary gene *traf3ip1* (also known as *ift54* or *elipsa*), which was shown to abolish all cilia at larval stages.^62^ This zebrafish mutant has been previously shown to display retinal defects at larval stages.^63^ In our study, we first determined the onset of ciliary defects in the *elipsa* mutant. We identified a progressive ciliary loss between the 10 somites stage and 30 hours postfertilization (hpf), allowing us to study specifically the role of cilia after the process of neurulation. Upon histological analysis, we observed that ciliary defects alter the overall brain morphology of zebrafish larvae, especially the cerebellum. We also identified an increased cell proliferation in the optic tectum, which is the visual processing center in zebrafish. Next, using transcriptomics, we revealed that genes and pathways involved in retinal function and cilia-mediated signaling, such as hedgehog signaling, are dampened by ciliary defects. Finally, we identified that the mutants have reduced light-evoked and ongoing neuronal activity in all brain areas, but do not display seizures. Taken together, our work identified that cilia are critical for brain development and physiology of neural circuits in zebrafish. This sets the stage for future analysis of ciliopathy models.

## Results

### Loss-of-function mutation of *traf3ip1 (elipsa)* leads to cilia defects in the developing brain following neurulation

To study the impact of ciliary loss on brain development, we selected a zebrafish mutant line, *elipsa* or *traf3ip1*^*tp49d*^,^62^ which was shown to lack primary and motile cilia at larval stages. We first aimed to identify the onset and penetrance of cilia loss in the *elipsa* mutant brain upon immunostaining of various developmental stages from 10 somites to 4 days post-fertilization (dpf) larvae. We used antibodies against acetylated tubulin and the ciliary protein arl13b, which labels both primary and motile cilia.^64^ To label motile cilia specifically, we used glutamylated tubulin as a marker as previously described.^48^^,^^50^^,^^65^ Upon staining with anti-acetylated tubulin at 10 somites stage, we found no major differences in cilia between control and mutants in the neural keel, developing optic vesicles (Figures S1A1, S1A2, S1B1, and S1B2) and in the left-right organizer (Kupffer’s vesicle) (Figures S1A3 and S1B3), suggesting that the maternal contribution of the gene prevents a ciliary phenotype at these early developmental stages.

**Figure 1:**
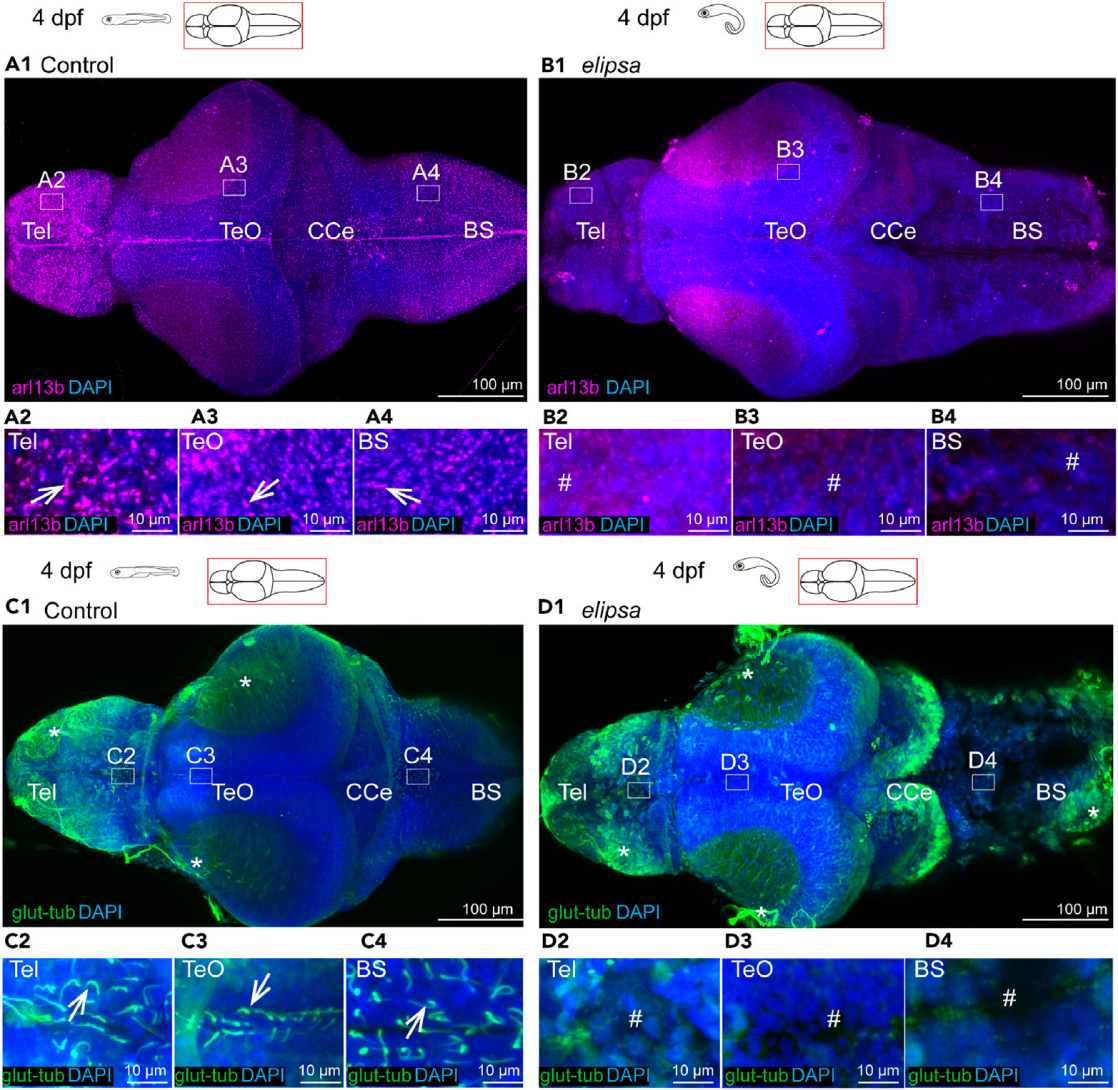
Loss of primary and motile cilia in the *elipsa* mutant larval brain (A1–A4 and B1–B4) Staining of dissected 4 dpf brains with arl13b antibody to stain all cilia in the brain, *n* = 8 controls and 9 mutants. A1 At 4 dpf, arl13b stained cilia were located all over the brain, represented in insets drawn in the telencephalon (A2), optic tectum (A3) and brainstem (A4). (B1) In the *elipsa* mutants, arl13b-expressing cilia were absent in the entire brain, as represented by insets drawn in the telencephalon (B2), optic tectum (B3), and brain stem (B4). (C1–C4 and D1–D4) Staining of dissected 4 dpf brains with glutamylated tubulin used to identify motile cilia, *n* = 5. (C1) At 4 dpf, single glutamylated tubulin-positive cilia were present in the forebrain choroid plexus (C2), on the dorsal roof and ventral part (C3) of the tectal/diencephalic ventricle and in the rhombencephalon choroid plexus (C4). (D1) In *elipsa* mutant, glutamylated tubulin-positive cilia were absent in the telencephalon (D2), optic tectum (D3), and brain stem (D4). Tel, Telencephalon; Teo, Optic Tectum; CCe, Corpus Cerebelli; BS, Brain stem. Cilia loss indicated by # symbol and nonspecific signal from glutamylated tubulin is represented by ∗ symbol. See also Figures S1 and S2.

Next, we investigated later time points of development following neurulation, from 30 hpf up to 4 dpf, when many mutants were still healthy and did not show major abnormalities beside their curved body axis and a mild heart edema. We noticed a total loss of arl13b and glutamylated-tubulin labeled cilia in the *elipsa* embryos at 30 hpf (Figures S2A1–S2B4), which was maintained at 2 dpf (Figures S2C1–S2F4) and 4 dpf (Figure 1). Notably, we observed that both primary cilia, expressing arl13b (Figures 1A1–1B4), and motile cilia, expressing glutamylated-tubulin (Figures 1C1–1D4), were defective in the entire brain at 2 dpf and 4 dpf. Taken together, our results reveal a progressive ciliogenesis defect in the brain of *elipsa* mutant after neurulation, allowing us to study the impacts of cilia on the development and physiology of the nervous system in an animal devoid of early neural tube defects.

### Cilia defects after neurulation lead to abnormal brain morphology and increase proliferation in the optic tectum

**Figure 2:**
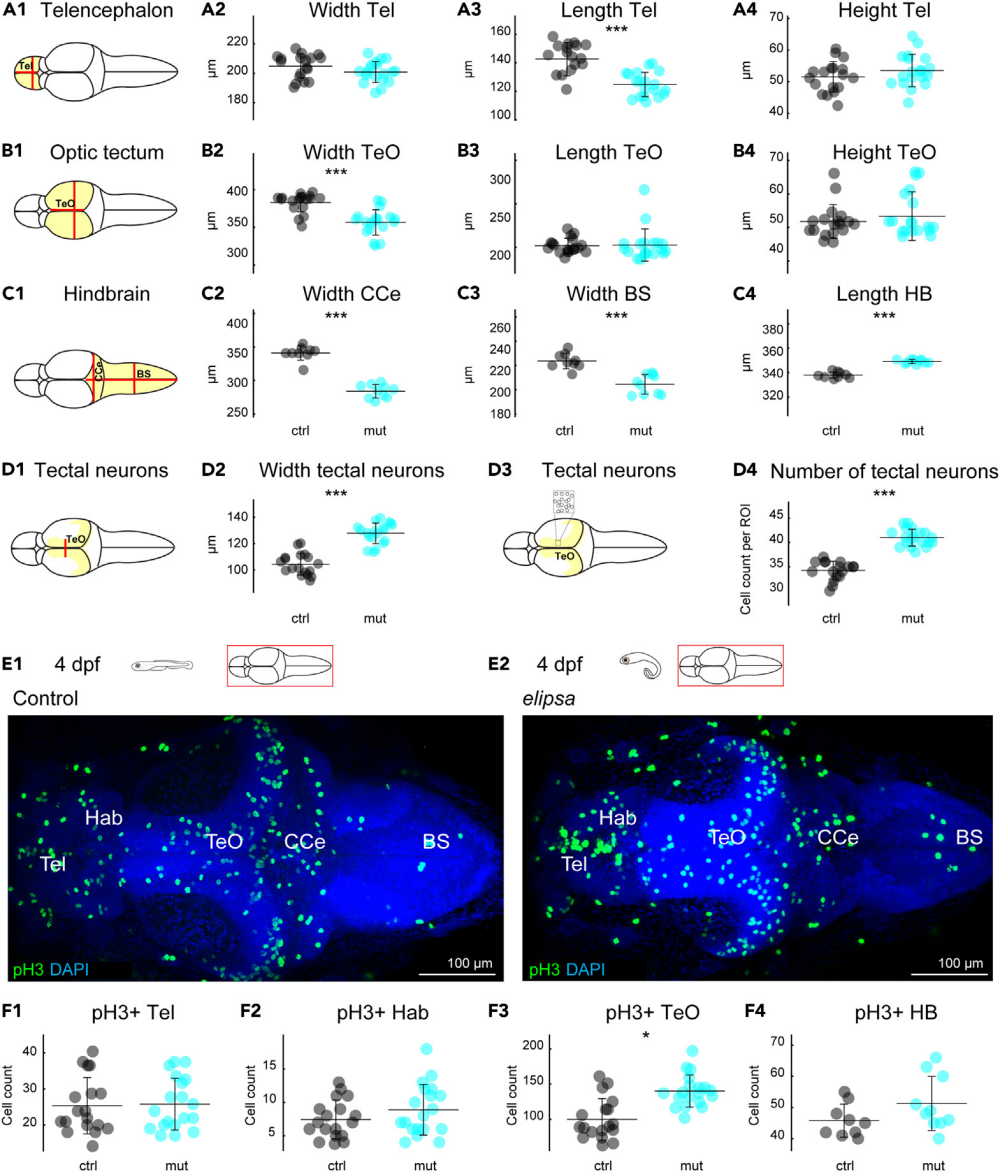
Cilia defects lead to abnormal brain size and alters cell proliferation in the optic tectum (A1–D4) Quantification of brain morphology of 4 dpf larval brains for control (black) and *elipsa* mutants (cyan). Schematic representation of the measurements for (A1) telencephalon, (B1) optic tectum, (C1) hindbrain regions and (D1) tectal neurons. Brain size was estimated by measuring the width, length, and height of telencephalon (A2, A3, A4), optic tectum (B2, B3, B4), width of CCe (C2), width of BS (C3), length of hindbrain (C4), and the width (D2) and number of neurons in a defined region of interest (D3) (ROI of 36 × 28μm) (D4) of tectal neurons. Controls are in black and *elipsa* mutants in cyan. *n* = 18 controls and 19 mutants for A2, A3, A4, B2, B3, B4, D2, D4. *n* = 9 controls and 10 mutants for C2, C3 and C4. (E1 and E2) staining for mitotic cells using an anti-pH3 antibody. (F1–F4) Cell count for pH3 positive cells (pH3+) in telencephalon (F1), habenula (F2), optic tectum (F3) and hindbrain (F4). ∗: *p* < 0.05, ∗∗∗: *p* < 0.001 according to Wilcoxon Rank-Sum test. Mean ± SD (standard deviation) is indicated on scatterplots. Tel, Telencephalon; Teo, Optic Tectum; BS, Brain stem; CCe, Corpus Cerebelli; HB, Hindbrain.

To identify the impact of cilia defects on brain development, we conducted various estimations of the brain size, including the width, length, and height of the telencephalon (Figures 2A1–2A4), optic tectum (Figures 2B1–2B4), and hindbrain (Figures 2C1–2C4). In our analysis we found a significant decrease in the length of the telencephalon (Figure 2A3), the width of the optic tectum (Figure 2B2), cerebellum (Figure 2C2) and brain stem (Figure 2C3). In contrast, the *elipsa* mutants displayed a significant increase in hindbrain length (Figure 2C4). Interestingly, while the overall width of the optic tectum was reduced, the thickness and density of the cellular layer in the tectum was increased (Figures 2D2 and 2D4) (see methods for details on quantifications). Notably, malformation of the hindbrain, especially the cerebellum, was apparent upon staining with glutamylated tubulin at 4 dpf (Figures 1C1–1D1).

To determine if the defects in brain size were due to a reduction in cell proliferation, we stained mitotic cells using a phosphorylated Histone H3 (pH3) antibody (Figures 2E1 and 2E2).^66^ We then quantified pH3 positive cells in distinct brain regions using confocal stacks spanning the entire brain (Figures 2F1–2F4). Our quantifications showed a significantly higher number of mitotic cells in the optic tectum in *elipsa* mutants (Figure 2F3) while we did not observe differences in cell numbers in other brain regions (Figure 2F1, 2F2, and 2F4). These results are consistent with our observations of increased cell density in the optic tectum in the *elipsa* mutants (Figure 2D4). Our findings suggest that cilia defects after neurulation disrupt normal brain development and results in an overall smaller brain with more pronounced malformations in the optic tectum and hindbrain.

### Cilia defects lead to morphological abnormalities of the cerebellum and reduced number of cerebellar Purkinje cells

**Figure 3:**
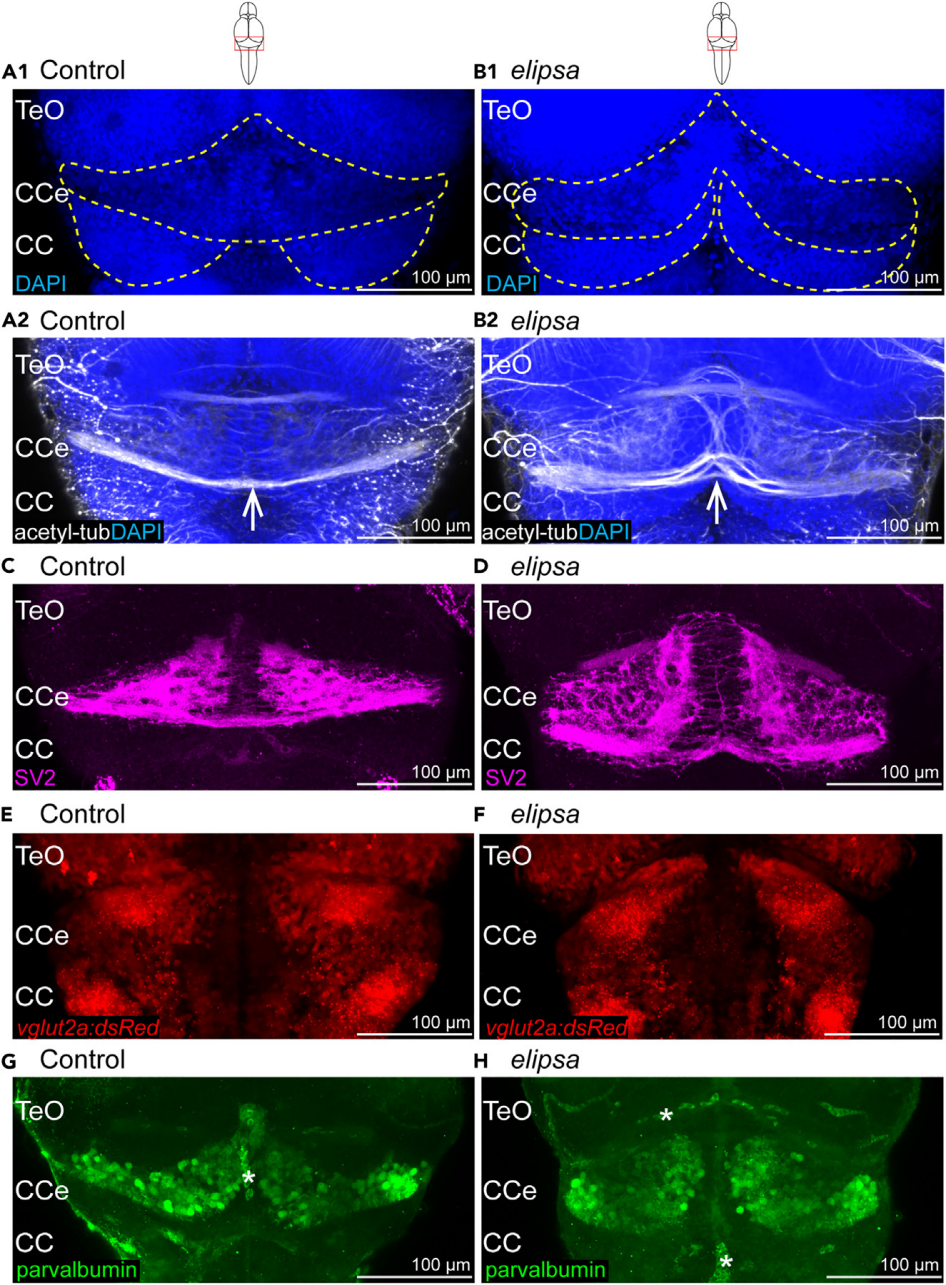
Cerebellar defects in 4 days old *elipsa* mutants (A1–B1) Cell nucleus stained using dapi in control (A1) and *elipsa* mutant (B1). Differences in morphology indicated by yellow lines. *n* = 7 controls and 7 mutants. (A2–B2) Larvae immunostained with anti-acetylated tubulin to stain axonal bundles (indicated with white arrow) in control (A2) and *elipsa* mutant (B2). *n* = 7 controls and 7 mutants. (C and D) Larvae immunostained with the presynaptic vesicle marker SV2 in control (C) and *elipsa* mutant (D). *n* = 6 controls and 6 mutants (E and F) Expression of glutamatergic cells in *Tg(vglut2a:dsRed)* transgenic zebrafish in control (E) and *elipsa* mutant (F). *n* = 8 controls and 8 mutants (G and H) Immunostaining of cerebellar Purkinje neurons using anti-parvalbumin antibody in control (G) and *elipsa* mutant (H). *n* = 10 controls and 8 mutants. ∗ indicates nonspecific signal from blood cells and vasculature. Number of Purkinje cells in control (mean ± standard deviation): 83.87 ± 5.22 cells, in mutants: 58 ± 6.8 cells, *p*-value 4,6965E-06 according to Student’s t test. Teo, Optic Tectum; CCe, Corpus Cerebelli; CC, Crista cerebralis.

Given the established role of cilia in cerebellar development^67^^,^^68^ and the abnormal glutamylated tubulin staining observed in the cerebellum (Figures 1C1-1D1), we further investigated the cerebellar anatomy using various histological and transgenic markers. First, using the nuclear stain dapi (Figures 3A1–3B1), we observed obvious cerebellar malformations in the *elipsa* mutant (Figure 3B1) in comparison to the controls (Figure 3A1). Next, we used an anti-acetylated-tubulin antibody to stain the axonal connections in the cerebellar region (Figures 3A2–3B2). Our results displayed abnormal axonal projections between the two cerebellar hemispheres in the mutants (Figure 3B2). Similar results were obtained using the presynaptic marker SV2 (Figures 3C and 3D). We next aimed to identify whether specific cell populations were particularly affected in the mutants, considering that the cerebellum is composed of both glutamatergic excitatory and GABAergic inhibitory neurons.^69^^,^^70^^,^^71^ We first assessed the excitatory glutamatergic neurons, which consist of mainly eurydendroid cells in addition to some granule cells,^70^ (Figures 3E and 3F) using the transgenic line *Tg(vglut2a:dsRed)*,^72^ but did not observe a major difference in the location of excitatory glutamatergic neurons despite the abnormal cerebellar morphology. In contrast, the number of parvalbumin-expressing inhibitory Purkinje cells (Figures 3G and 3H) was significantly reduced in the mutant animals (Figure 3H). Altogether, our findings show that cilia defects in the *elipsa* mutant disrupt cerebellar development and lead to malformations and mispatterning.

### Transcriptomic analysis identifies differentially regulated genes involved in phototransduction and brain development

**Figure 4:**
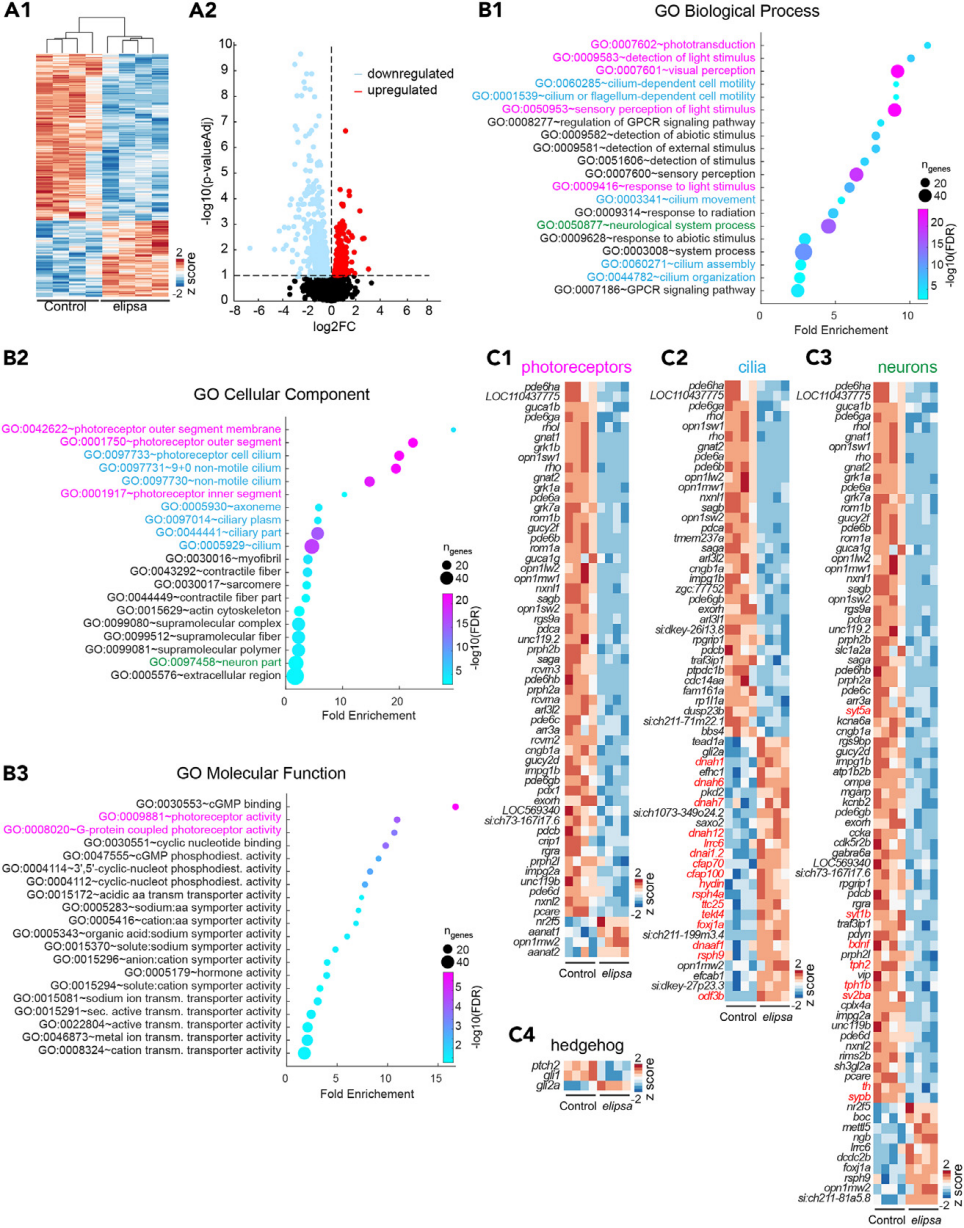
Transcriptomic analysis reveals altered expression of genes involved in phototransduction (A1) Heatmap of expression of differentially expressed genes (DEGs) from control and *elipsa* 4dpf whole larvae (*n* = 4 RNA preparation obtained from circa 30 elipsa and sibling controls) shown as *Z* score of log2-normalized read counts. Increased expression is represented with red and decreased expression with blue. A total of 702 (220 upregulated and 482 downregulated) DEGs were identified based on a threshold of *p*-value adjusted of 0.1. (A2) Volcano plot showing the DEGs in *elipsa* as compared with control. The horizontal dotted line indicates the adjusted *p*-value of 0.1. The vertical dotted line separate upregulated (red) and downregulated (blue) DEGs. (B) Top 20 Gene Ontology (GO) biological process (B1) cellular component (B2) and molecular function (B3). Genes associated with GO terms related to phototransduction (magenta), cilia (blue) and neuronal process (green) are represented in (C1–C3). (C) Heatmaps showing expression of DEGs (represented as *Z* score of log2-normalized read counts) associated with (C1) photoreceptor and light detection GO terms (magenta highlights in B1-B3), (C2) cilia GO terms (blue highlights in B1-B2), (C3) neuronal process (green highlight in B1-B2), and (C4) hedgehog signaling (receptors *smo* and *ptch1-2* and transcription factors *gli1-3*). Red highlights show genes associated with motile cilia function in (C2) and genes of interest in (C3). See also Figure S3, Tables S1 and S2.

To explore the impact of cilia defects on gene expression, we performed RNA-sequencing of whole 4 dpf larvae. We identified a total of 702 differentially expressed genes (DEGs), comprising 220 upregulated DEGs and 482 downregulated DEGs (Figures 4A1, 4A2, and S3; Table S1), between control and *elipsa* larvae. We next classified the genes into different Gene Ontology (GO) terms consisting of GO biological process, GO cellular component, and GO molecular function (Figures 4B1–4B3, and Table S2). The most significant GO terms were related to phototransduction, photoreceptors and cilia (Figures 4B1–4B3). Genes associated with photoreceptors and phototransduction GO terms were mostly downregulated (Figure 4C1), whereas genes associated with cilia-related GO terms were either downregulated or upregulated (Figure 4C2). In fact, most genes associated with motile cilia function, e.g., the master regulator of motile ciliogenesis *foxj1a* and some of its target genes,^30^^,^^73^ were upregulated (Figure 4C2, red highlight), suggesting a potential genetic compensation mechanism as observed in the *ccdc103* (also known as *smh*) and *rpgrip1l* mutants.^51^^,^^74^ Among genes associated with the GO terms “neurological system process” and “neuron part”, we identified that genes encoding for the grow factor brain-derived neurotrophic factor (*bdnf*),^75^ various synaptic proteins (*synaptotagmin*, *synaptic vesicle glycoprotein 2b*, *synaptophysin b*) and enzymes involved in serotonin and catecholamine production (*tyrosin* and *tryptophan hydroxylase*) were downregulated (Figure 4C3, red highlights). Finally, genes involved in common cilia-related signaling pathways especially the sonic hedgehog pathway were differentially regulated suggesting a dampened sonic hedgehog pathway^15^^,^^17^ (Figure 4C4). Overall, our transcriptomics findings highlight a potential retinal defect in *elipsa* mutants and the influence of cilia defects on specific signaling pathways like sonic hedgehog.

### Cilia defects in *elipsa* larvae result in morphological and physiological defects in the photoreceptor layer

**Figure 5:**
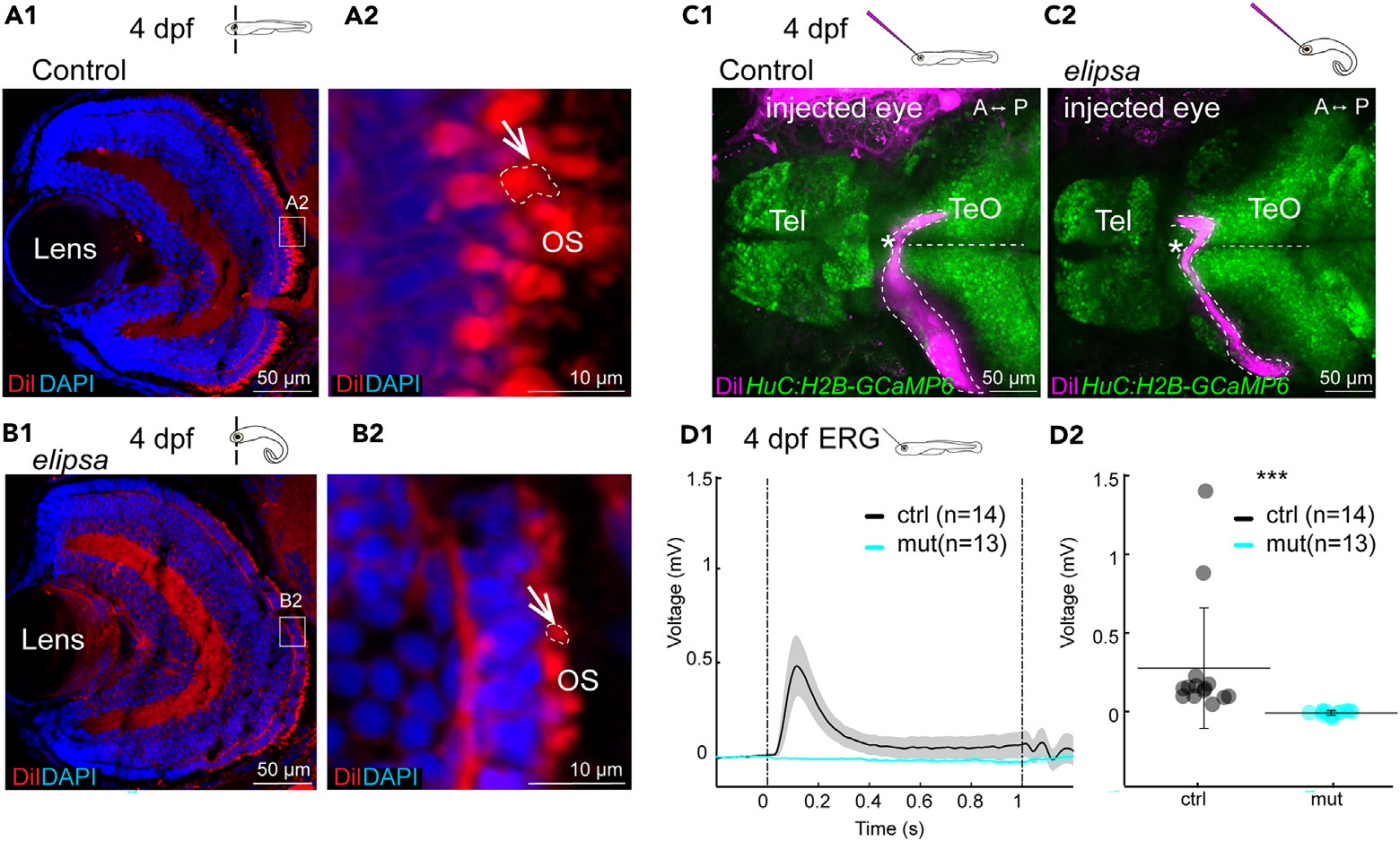
*elipsa* mutants display morphological defects of the photoreceptor outer segments and no retinal electrical activity (A1–B2) DiI staining of 4 dpf retina cryosection to stain the outer segments. Section through the whole retina or the photoreceptor layer of a representative control (A1-A2) and *elipsa* (B1-B2). Shortened outer segments (OS) are indicated using arrow and dashed lines. *n* = 10 controls and 12 mutants. (C1-C2) DiI injection into the eye at 4dpf to stain the axonal connections (represented in dotted lines) which cross the midline and innervate the contralateral optic tectum. Here is shown a representative control (C1) and *elipsa* (C2). Note that in the *elipsa* mutant the axonal tract goes posterior prior to returning to the optic chiasm. ∗ indicates region of optic chiasm. *n* = 9 controls and 8 mutants. See Figure S4 for additional examples. (D1-D2) Electroretinography (ERG) recordings in a 4 dpf retina. D1 Average response of electrical activity (+/− standard error of the mean as the shaded region) to 1 s light stimulation for control (black) and mutant (cyan). D2 Average electrical responses for the 200 msec following the light ON stimulus for all control fish (black) and *elipsa* mutant (cyan) showing no activity in the mutant. ∗∗∗: *p* < 0.001 according to Wilcoxon Rank-Sum test. Mean+/− standard deviation is indicated on scatterplots. *n* = 14 controls and 13 mutants. Tel, Telencephalon; Teo, Optic Tectum. See also Figure S4.

Since our transcriptomic analysis revealed downregulation of genes involved in phototransduction implying possible defects with the retina, we next sought to assess the morphology and physiological activity of the mutant’s retina. Notably, primary cilia were shown to play a crucial role in the formation and maintenance of photoreceptor outer segments in the vertebrate retina.^9^ In line with this, prior work in the *elipsa* mutant identified a progressive degeneration of the photoreceptor outer segments at 5 dpf, with no morphological difference at 3 dpf.^63^ Here, we aimed to analyze the retinal phenotype at 4 dpf when mutant larvae remain relatively healthy beside their curved body axis. To this end we prepared retinal cryosections of 4 dpf control and *elipsa* larvae (Figures 5A1 and 5B1) and stained them using the lipophilic DiI dye. In comparison to the control larvae which had intact elongated outer segments (Figure 5A2), the outer segments of the *elipsa* larvae were shortened (Figure 5B2). Besides the photoreceptors, we did not observe major defects in the layering of the retina in both groups. Yet, we identified abnormal axonal projections from the retinal ganglion cells to the tectum near the optic chiasm in larvae with impaired cilia upon DiI injections (Figures 5C1, 5C2, and S4A1–S4B6). Although all *elipsa* mutants displayed correct contralateral projections, the retinal ganglion cells commonly had circumvoluted tracts at the region of the optic chiasm (Figure 5C2). Since we did not analyze in detail the axonal projection within the tectal neuropil, we cannot exclude mispatterning of the retinal ganglion axonal terminals. Next, we investigated visual function by electroretinography (ERG) in 4 dpf larvae (Figures 5D1 and 5D2), using a train of 1-second-long blue light stimuli. We observed a complete absence of ERG signals in all analyzed *elipsa* as compared to controls. Taken together our results identified that cilia loss in the *elipsa* leads to morphological defects and loss of electrical activity in the retina already at 4 dpf.

### Reduced photic-induced neural activity in all brain regions of cilia-deficient larvae

**Figure 6:**
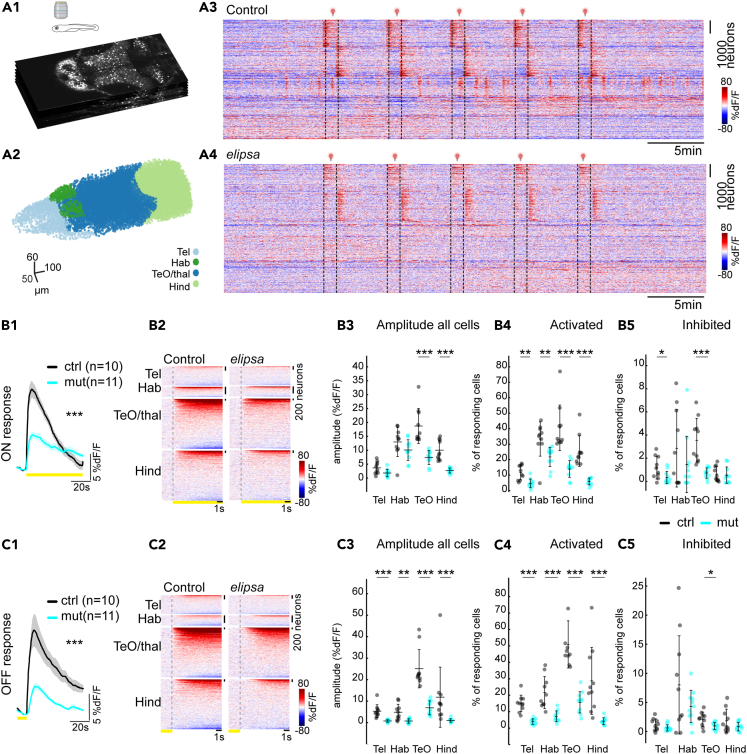
Reduced photic-induced neural activity in the brain of *elipsa* mutants (A1) Optical sections of multiplane recording of a transgenic zebrafish larva expressing nuclear GcaMP6s in all neurons *Tg(elavl3:H2B-GcaMP6s)*^*jf7Tg*^. (A2) Segmented nuclei from different brain regions, which were identified based on anatomical landmarks, are color coded. (A3-A4) Neuronal activity represented as change of fluorescence (dF/F) in one representative control and mutant larvae. Traces were sorted based on their activity using k-means clustering (warm color represents higher calcium signals). (B1, C1) Neural responses to light stimulation for control (black) and mutant (cyan) averaged over 5 trials of photic stimulations for ON (B1) and OFF (C1) response conditions (+/− standard error of mean as the shaded region trace). (B2, C2) Activity of neurons per brain regions during ON (B2) and OFF response (C2) for representative examples (warm color represents higher calcium signals). (B3, C3) Average amplitude of all cells during ON (B3) and OFF (C3) response for control (black) and mutant (cyan), amplitude was significantly reduced in TeO and Hind regions for ON response and all brain regions for OFF response. (B4, C4 and B5, C5) % of cells that are activated during ON (B4) and OFF response (C4) and inhibited during ON (B5) and OFF response (C5). ∗: *p* < 0.05, ∗∗: *p* < 0.01, ∗∗∗: *p* < 0.001 according to Wilcoxon Rank-Sum test. Mean+/− standard deviation is indicated on scatterplots. *n* = 10 controls and 11 mutants, control (black) and mutant (cyan). Tel, Telencephalon; TeO, Optic Tectum; BS, Brainstem; Hind, Hindbrain.

To identify the impact of cilia defects on neuronal activity, we performed volumetric two-photon calcium imaging in 4 dpf *elipsa* larvae expressing the nuclear calcium indicator H2B-GCaMP6s in all differentiated neurons (Figure 6A1).^76^ We performed 40 min-long recordings consisting of spontaneous activity, followed by a series of photic stimulations that measure visual responses and can trigger seizure-like activity.^77^ We recorded 8 planes, used five light flashes of 1 min each and collected data from four specific brain areas: the telencephalon, optic tectum/thalamus, habenula, and hindbrain (Figure 6A2). Upon sorting cells based on k-means clustering, we observed distinct clusters of neurons exhibiting either enhanced, reduced or unchanged activity during photic stimulations, as shown by the representative examples (Figures 6A3 and 6A4). To quantify neuronal response, we averaged the 5 photic stimulations and analyzed the neuronal activity during the light ON and OFF responses separately. We first examined the average activity of all neurons and found significantly reduced response for both ON (Figures 6B1–6B5) and OFF (Figures 6C1–6C5) conditions. In the controls, the optic tectum and hindbrain displayed the strongest responses to light flashes, while the telencephalon and habenula displayed comparatively minimal responses (Figures 6B2 and 6C2). By computing the response amplitude for all cells in both light ON and OFF conditions (Figures 6B3 and 6C3), we observed reduced neuronal activity in most brain areas of the *elipsa* mutants. Notably, the tectum and hindbrain neurons exhibited reduced amplitudes for the light ON response condition (Figure 6B3), while all brain regions displayed lower amplitudes for the OFF condition (Figure 6C3). We then identified whether cells were generally less active or whether there were fewer responding cells. To determine the responding cells, we analyzed 10 s after the light stimulus and categorized cells with a mean amplitude greater than 2 standard deviations of the baseline as responding (as described in the methods). Our findings revealed a significant reduction in the number of activated cells across all brain regions for both light ON (Figure 6B4) and OFF conditions (Figure 6C4). Moreover, we noted significant decrease in the number of inhibited cells in the telencephalon and optic tectum for the light ON condition (Figure 6B5) and only in the optic tectum for the light OFF condition (Figure 6C5).

We previously identified that photic stimulation serves as a good strategy to detect hyperexcitability and trigger seizures in epilepsy models.^77^ We did not observe any epileptic seizures in the 11 mutant animals analyzed, suggesting that ciliary loss does not lead to the occurrence of seizure in the *elipsa* mutants at 4 dpf.

Taken together, our results using photic stimulation show that mutant animals exhibit reduced light-evoked neuronal activity in the entire brain, which aligns well with their retinal dysfunction. Interestingly, we measured residual neuronal activity throughout the brain of *elipsa* mutants despite the absence of ERG responses, suggesting that non retinal light-sensitive pathways remain at least partially active upon ciliary defects.

### Reduced spontaneous activity upon cilia defects in *elipsa* mutant larvae

**Figure 7:**
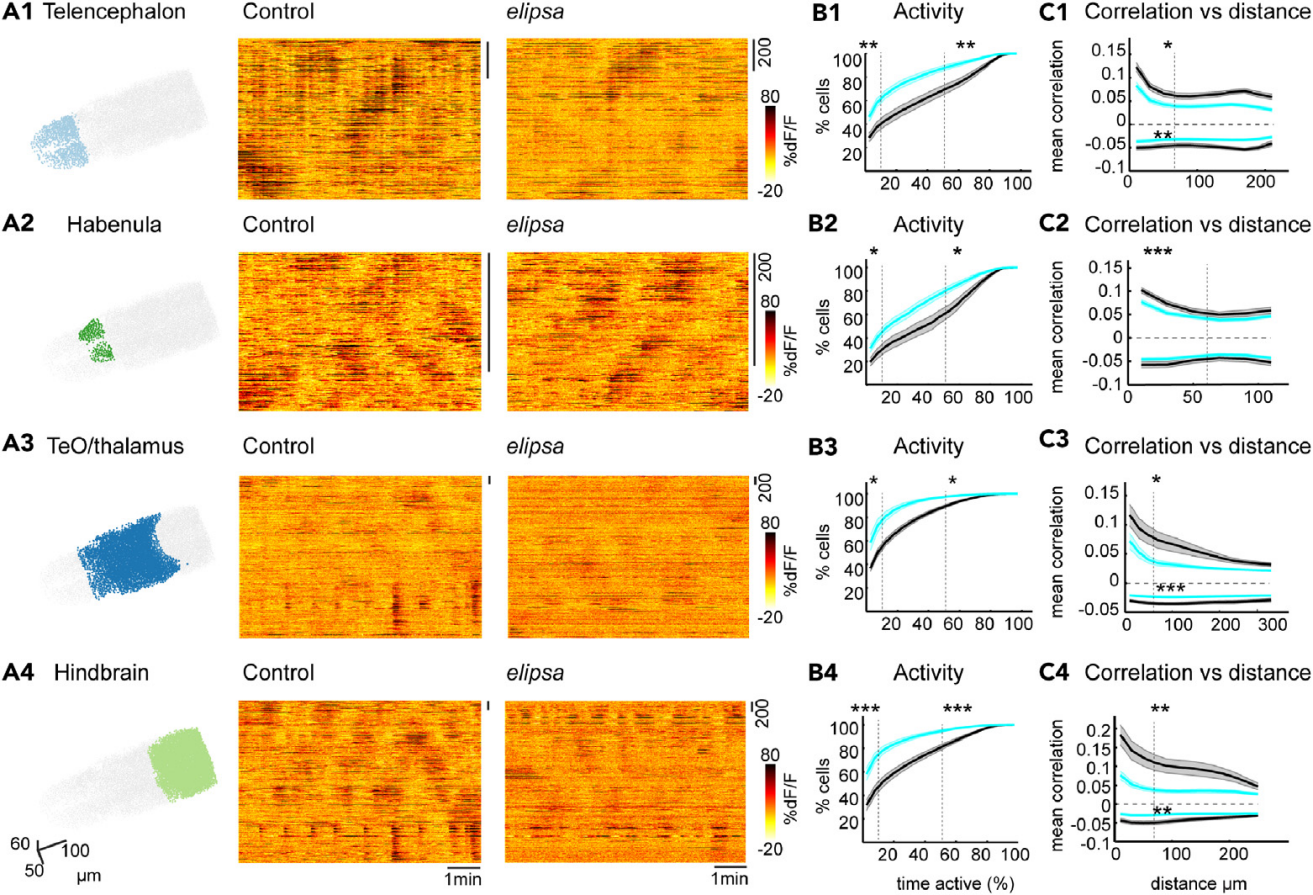
Reduced ongoing spontaneous activity and correlation in the *elipsa* mutants (A1–A4) Three-dimensional representation of segmented neurons and their activity for one representative control and *elipsa* for the Telencephalon (A1), Habenula (A2), TeO/thalamus (A3) and Hindbrain (A4). Dark color represents higher dF/F signals. (B1–B4) Average cumulative frequency distribution graphs showing the percentage of activity of neurons in Telencephalon (B1), Habenula (B2), TeO/thalamus (B3) and Hindbrain (B4), for control (black) and *elipsa* (cyan). (+/− standard error of mean as the shaded area). Dotted lines indicate activity below 10% and above 50%. (C1–C4) Mean Pearson’s correlation versus distance graphs showing positive and negative correlation between cells in Telencephalon (C1), Habenula (C2), TeO/thalamus (C3) and Hindbrain (C4), for control (black) and mutant (cyan). Significance tests for the correlation were computed for cells located within 60 μm indicated by the dotted line on the X axis. ∗: *p* < 0.05, ∗∗: *p* < 0.01, ∗∗∗: *p* < 0.001 according to Wilcoxon Rank-Sum test. *n* = 10 controls and 11 mutants. See also Figure S5.

We next sought to investigate the impact of ciliogenesis defects on spontaneous brain activity. To this end, we quantified ongoing brain activity in control and *elipsa*. As shown in a representative example where neuronal activity was sorted based on their similarities using the rastermap,^78^ *elipsa* mutants showed generally less activity in all brain regions (Figures 7A1–7A4). Next, we measured the activity of cells by quantifying the percentage of time they spent above a threshold (see STAR Methods) and generated an average cumulative frequency distribution for each brain regions. Our analysis showed that significantly fewer cells were highly active (active more than 50% of the time) in all brain regions in mutant animals (Figures 7B1–7B4 and S5A1–S5D2). Concurrently, more cells were inactive (active less than 10% of the time) in the mutant (Figures 7B1–7B4 and S5A1–S5D2). Furthermore, we investigated whether cells were differently correlated with each other. To this end we calculated the mean Pearson’s correlations as a function of the distance between pairs of cells within distinct brain regions. Our results identified that neurons that are closer to each other have higher positive correlations. Notably, we calculated mean correlations of 0.1–0.2 depending on the brain region, which are consistent with previous reports.^79^^,^^80^^,^^81^ Our analysis also revealed significantly less positive and negative correlation between nearby cells in the *elipsa* mutants (Figures 7C1–7C4 and S5E1–S5H2). Altogether our results reveal that in absence of cilia neurons are less active and less correlated with each other, thereby emphasizing an important role of cilia in establishing functional neural networks in the zebrafish brain.

## Discussion

Cilia have been associated with a wide variety of functions in the developing brain, from neuronal proliferation and differentiation to axonal pathfinding and neuromodulation. Yet, how alterations in cilia-related neurodevelopmental processes impact brain activity have remained understudied. Moreover, despite zebrafish being a very common and powerful model to study cilia dysfunction, there have been limited reports on the function of cilia in the zebrafish brain. In our study, we took advantage of the small size of the zebrafish brain and its genetic toolbox to show that ciliary defects after neurulation alter brain development and the establishment of functional neural circuits using whole brain calcium imaging.

In our study, we chose specifically a zebrafish mutant line, *elipsa*/*traf3ip1* encoding for ift54, which has been studied earlier in the context of the retina, auditory hair cells, olfactory sensory neurons, spinal canal, brain ventricles, and pronephros.^48^^,^^62^^,^^63^^,^^82^^,^^83^ In agreement with these prior works, we identified cilia defects in the brain of *elipsa* mutants at stages following neurulation. Notably, we did not observe obvious ciliary defects in earlier developmental stages when the neural keel and Kupffer’s vesicle are established. The presence of cilia in the mutants at the 10 somites stage is most probably due to the maternal contribution of transcripts. Since we discovered that the mutant embryos start to show defects between 13 hpf and 30 hpf, our observations indicate that ciliogenesis initially takes place but is subsequently impaired during brain development and explain the absence of neural tube patterning defects in the *elipsa* mutant brains.

To date, cilia defects were mainly shown to lead to brain malformations in rodent models and human ciliopathy patients. These include abnormalities in the organization and connectivity of neurons and altered neuronal migration in various brain structures including the cortex, cerebellum, and hippocampus.^10^^,^^19^^,^^84^^,^^85^ In our study, we found that impairment of ciliogenesis had significant consequences for brain development also in zebrafish. We observed not only size differences for the telencephalon, optic tectum, and hindbrain but also malformations in the cerebellum along with disrupted axonal tracks and reduction in Purkinje cell numbers in the cerebellar regions. These findings align well with the phenotype of patients affected by Joubert’s syndrome^56^^,^^57^ which displays absence or underdevelopment of the cerebellar vermis as well as a malformed brain stem.^59^^,^^60^^,^^86^^,^^87^^,^^88^^,^^89^ Of note, cerebellar defects have only been described in a single zebrafish mutant so far, carrying a loss of function allele in the Joubert gene *arl13b*.^71^^,^^86^^,^^90^ Notably, our results in the *elipsa* mutants are similar to the ones reported for the Joubert gene *arl13b* mutant zebrafish.^90^ By characterizing cerebellar malformation in the *elipsa* mutant, our findings are therefore opening new avenues for understanding cilia-related control of cerebellar development in Joubert’s syndrome using zebrafish as model system.

Since cilia serve as vital structures for the regulation of the cell cycle,^14^^,^^91^^,^^92^ we aimed to identify if the reduced brain size in our cilia mutant related to problems in cell proliferation. We did not observe any reduction in number of dividing cells but rather a significantly higher number of dividing cells in one specific brain area, the optic tectum. Some neural progenitors in the optic tectum are of neuroepithelial characteristics in contrast to the radial glia of the telencephalon.^93^^,^^94^^,^^95^ Therefore, it is possible that distinct types of neural progenitors are differentially affected by ciliary dysfunction and call for future work on identifying the molecular mechanisms underlying these differences. Furthermore, although the number of dividing cells and tectal neurons were increased in the mutants, we still noticed a significant smaller tectum. Previous work in the *elipsa* mutant showed an increased cell death in the retina at 4 dpf.^63^ Given these results it is possible that apoptosis is also increased in the brain of the *elipsa* mutant which could explain the reduced brain size.

Our transcriptomic analysis mainly uncovered genes linked to phototransduction and the formation of the outer segments of the retina. These results corroborated well with the photoreceptor abnormalities that we characterized, including a significant reduction in the size of outer segments and loss of retinal electrical activity. Our findings are consistent with previous work,^63^ which described the impact of the *elipsa* mutation on the structure and function of the retina at 5 dpf. It is important to note that despite observing a fully penetrant retinal dysfunction at 5 dpf, this study did not report any apparent morphological abnormalities at 3 dpf,^63^ supporting the fact that cilia-related retinal defects are usually progressive and worsens over time. We now provide further evidence that the retina is already affected at 4 dpf, a developmental stage when larvae remain relatively healthy to be analyzed in depth for neuronal function.

To analyze the impact of ciliary defects on brain physiology, we used volumetric two-photon calcium imaging and measured spontaneous and light-induced neuronal activity. In zebrafish, light stimulation elicits neuronal responses in various brain regions. These include the visual center known as the optic tectum,^96^ the thalamus,^97^ which receive direct input from the retinal ganglion cells, and their downstream projections to the habenula,^98^^,^^99^ telencephalon and hindbrain.^97^ In line with retinal defects, we observed a significant decrease in the light response across all brain regions in *elipsa* larvae. Notably, we did not observe a complete loss of light-induced neuronal activity in the *elipsa* mutants. This could be related to a potential residual photoreceptor activity in the mutant, which were too small to be detected by ERG, or non-retinal light responses. The pineal gland, a ciliated organ located dorsally above the telencephalon,^100^^,^^101^ may be involved, although one could expect that cilia loss would affect the pineal responses too. Alternatively, it has been reported that specific population of neurons express light-responsive opsins, which regulate animal behavior.^102^^,^^103^^,^^104^ These neurons may therefore be involved in the light responses that remained in the *elipsa* mutants. One prior report identified that *elipsa* morphants^105^ displayed altered proliferation of retinal progenitors and survival of RGCs. Therefore, it is possible that the reduced light-evoked neuronal activity in the *elipsa* mutants stems from an accumulation of developmental defects, loss of outer segments and reduced photoreceptor activity.

Our investigation into spontaneous activity^79^^,^^80^^,^^106^ revealed notable differences in the *elipsa* mutants including significant reduction in the number of active cells and correlation between cells. These differences can stem from the neurodevelopmental defects that we have observed. Since spontaneous activity can play a role in maturation of functional circuits, and refinement of axonal projections,^107^^,^^108^ it is possible that the reduced spontaneous activity further exacerbates defects in neuronal development and gene expression. Altered spontaneous activity in the brain is observed in various conditions, including neurodevelopmental disorders, neurodegenerative diseases, or brain injuries, which usually manifest with intellectual disabilities or cognitive impairments.^109^^,^^110^ Notably, ciliary dysfunction in Joubert and Bardet-Biedl syndromes is commonly associated with intellectual disabilities,^54^^,^^55^^,^^58^^,^^61^ suggesting that it may originate from altered spontaneous activity. Primary cilia have been associated with neuronal migration, axon guidance and synapse formation.^10^^,^^20^^,^^21^^,^^22^^,^^23^^,^^24^^,^^25^^,^^26^^,^^27^ Alterations in these processes may collectively explain the reduced spontaneous activity that we observed in *elipsa* mutants. Overall, while the precise link between cilia and spontaneous activity in the brain is not fully established, future experiments manipulating cilia dynamics in specific neurons will be able to uncover the specific molecular mechanisms controlling cilia-dependent brain activity.

Ciliopathy syndromes including Joubert and Bardet-Biedl syndrome are associated with epilepsy,^54^^,^^55^^,^^61^ which is a neurological disorder characterized by recurrent seizures. The exact mechanisms underlying the association between ciliopathies and epilepsy are not fully understood and may vary depending on the specific syndrome. While our study revealed cilia-related neurodevelopmental defects, we did not observe any spontaneous or light-evoked epileptic seizures in the *elipsa* mutant larvae. This may be related to the analyzed developmental stage, 4 dpf, which may be too early to detect seizure-like activity. Indeed, while some zebrafish epilepsy models, including the mutant carrying a loss-of-function mutation in the glutamate transporter *eaat2a*, were shown to already display seizures at 5 dpf,^111^ other models develop seizure only at the juvenile stages like the GABA A receptor mutants.^112^^,^^113^ Alternatively, one could argue that reduced retinal activity could prevent light-induced hyperexcitability in the *elipsa* mutant. Yet, the *eaat2a* mutants, which also show reduced retinal activity,^114^ display strong photic-induced epileptic seizures.^77^^,^^111^

In summary, our results demonstrate that cilia defects post neurulation have impacts on the development of functional neural circuits and lead to altered neuronal activity in the larval zebrafish brain. We are confident that this study opens avenues to study the molecular mechanisms underlying cilia-related brain dysfunction in ciliopathy models and identify potential therapeutic targets.

### Limitations of the study

Due to the limited availability of antibodies labeling cilia in zebrafish, our analysis of cilia defects in neurons relied on a single marker, namely arl13b. A loss of arl13b signal in cilia does not obligatorily mean that the *elipsa* mutants lack all cilia in neurons.^115^ Yet, given the known role of *traf3ip1/ift54* in ciliogenesis, the likelihood of cilia loss is high. Future work using additional markers or electron microscopy will be able to identify potential ciliary remnants or structural defects in the *elipsa* mutant.

Another limitation of our study was that the transcriptomic analyses of *elipsa* mutant were performed on whole larvae and not specifically on the brain or defined cell types. Thus, it was not possible with our approach to obtain a precise profile of differentially expressed genes in specific cell types. However, this was unlikely to have had a major impact in our conclusions since we focused on genes related to overall brain development and signaling.

In addition, all data reported in our study were obtained at larval stages up to 4 dpf. We could unfortunately not record data from older animals since mutant zebrafish with ciliogenesis defects, including *elipsa* mutant, typically display a curled tail and do not survive to adulthood. Recent work revealed that the Joubert syndrome gene *cc2d2a* mutant zebrafish displayed locomotory and postural defects,^71^ hinting at underlying abnormalities in neuronal activity and circuit function in this mutant. Future work using cell-specific manipulation of cilia will be critical to pinpoint the precise function of cilia in different cell types of the brain. Besides, a more detailed analysis of the glutamatergic populations and the connectivity of neuronal populations in the cerebellum is needed to better understand the impact of cilia defects on cerebellar development and physiology in the zebrafish.

## STAR★Methods

### Key resources table

**Table:** 

REAGENT or RESOURCE	SOURCE	IDENTIFIER
**Antibodies**		

Mouse monoclonal glutamylated tubulin (GT335)	Adipogen	Cat#AG-20B-0020-C100; RRID: AB_2490210
Mouse anti SV2 antibody	Developmental Studies Hybridoma Bank	Cat# 2315387; RRID:AB_2315387
Mouse anti parvalbumin antibody	Chemicon	Cat# MAB1572; RRID:AB_2174013
Rabbit Arl13b antibody	Zhaoxia Sun Lab	Duldulao et al., 2009^64^
Anti-acetylated tubulin antibody	Sigma	Cat#T7451; RRID:AB_609894
Rabbit p-Histone H3 Antibody	Santacruz	Cat#sc-8656-R; RRID:AB_653256
Alexa fluor plus Goat anti rabbit 555	Thermo Fisher Scientific	Cat#A32732; RRID:AB_2633281
Alexa fluor plus Goat anti mouse 555	Thermo Fisher Scientific	Cat#A32727, RRID:AB_2633276
Alexa fluor plus Goat anti mouse 647	Thermo Fisher Scientific	Cat#A32728; RRID:AB_2633277

**Chemicals, peptides, and recombinant proteins**		

DAPI (4',6-Diamidino-2-Phenylindole, Dihydrochloride)	Thermo Fisher Scientific	Cat#D1306; RRID:AB_2629482
Trichloroacetic Acid	Baker analyzed	Cat#10385960
Microamp optical 96 well reaction plate (Applied biosystems)	Thermo Fisher Scientific	Cat#N8010560
Phosphate buffered saline	Thermo Fisher	Cat#BR0014G
Triton X-100	Merck	Cat# 1086031000
Bovine Serum Albumin (BSA)	PanReac Applied Chem	Cat#A1391
Dimethyl sulfoxide (DMSO)	Sigma	Cat#D8418
Glycerol	VWR	Cat#24387.292
Acetone	VWR	Cat#20066.296
Formaldehyde solution (PFA)	Sigma	Cat#F8775-25ml
MS-222	Sigma	Cat#E10621-60G
Proteinase K from tritirachium album	Sigma	Cat#P2308-25MG
Tris	Sigma	Cat#252859-100G
Alpha-bungarotoxin	Invitrogen	Cat#B1601
Agarose	Thermo Scientific	Cat#P17850
Sucrose	Invitrogen	Cat# 15503022
Dil stain (1,1’-Dioctadecyl-3,3,3’,3’-Tetramethylindocarbocyanine Perchlorate (‘DiI’; DiIC_18_(3)))	Invitrogen	Cat#D282
ProLong Gold Antifade Mountant	Invitrogen	Cat#P36930 (10 ml)
Trizol	Invitrogen	Cat# 15596026
Rneasy mini kit	Qiagen	Cat#74104 (50 reactions)
Rnase-Free Dnase Set	Qiagen	Cat#79254 (50 reactions)
Ethanol (molecular grade)	Merck	Cat#1.00983
Ultra LMP agar	Invitrogen	Cat#16520-100
Thin wall capillary 1 mm	World precision instruments	Cat#TW100F-4 (with filament)
Filter paper	VWR	Cat#516-0848
Flurodish	VWR	Cat# FD35PDL-100
Glass capillary	WPI	Cat# TW100F-4

**Critical commercial assays**		

KASP Assay	LGC genomics	NA

**Deposited data**		

Raw confocal stack, two-photon activity traces, electroretinograms and codes used to generate figures	This study	NIRD research data archive: https://doi.org/10.11582/2024.00035
RNA sequencing raw and processed data	This study	GEO: accession number GSE244171

**Experimental models: Organisms/strains**		

*Elipsa traf3ip1*^*tp49d*^	Omori et al., 2008^62^	RRID:ZFIN_ZDB-ALT-980413-466
*Tg(elavl3:H2B-GCaMP6s)*^*jf7Tg*^	Vladimirov et al., 2014^76^	RRID:ZFIN_ZDB-ALT-150916-4
*Tg(vglut2a:dsRed)*	Miyasaka et al., 2009^72^	RRID:ZFIN_ZDB-ALT-100505-2

**Software and algorithms**		

ImageJ/Fiji	Schindelin et al., 2012^117^	

**Other**		

Pressure injector	Eppendorf	Femtojet 4i
Confocal microscope	Zeiss	Examiner Z1; Olympus Fluoview
Two photon microscope	Scientifica	NA
Step One Real Time PCR system	Thermofisher	Cat#4376357
Sutter Laser puller	Sutter	Model P-2000
Cryostat NX 70	Thermo Scientific	NA

### Resource availability

#### Lead contact

Further information and requests for resources and reagents should be directed to and will be fulfilled by the lead contact, Nathalie Jurisch-Yaksi (nathalie.jurisch-yaksi@ntnu.no).

#### Materials availability

This study did not generate new unique reagents.

#### Data and code availability

• RNA sequencing data have been deposited on the GEO repository and are publicly available with the accession number GSE244171. All raw confocal, two-photon and electroretinogram data are publicly available on NIRD research data archive at this link https://doi.org/10.11582/2024.00035.
• All codes to plot the figures and analyze two-photon data are publicly available on NIRD research data archive at this link https://doi.org/10.11582/2024.00035.
• Any additional information required to reanalyze the data reported in this paper is available from the lead contact upon request.

### Experimental model and study participant details

Zebrafish larvae were used for this study. The animal facilities and maintenance of the zebrafish, *Danio rerio*, were approved by the NFSA (Norwegian Food Safety Authority). All the procedures were performed on zebrafish were in accordance with the European Communities Council Directive, the Norwegian Food Safety Authorities. The larval and adult zebrafish were reared according to standard procedures of husbandry at 28.5°C, in 3.5 L tanks in a Techniplast Zebtech Multilinking system at constant pH 7 and 700 μSiemens, at a 14:10 hr light/dark cycle to mimic optimal natural breeding conditions. Larvae were maintained in egg water (1.2 g marine salt and 0.1% methylene blue in 20 L RO water) from fertilization to 3 dpf and subsequently in AFW (1.2 g marine salt in 20L RO water). For our experiments, the following fish line were used *elipsa/traf3ip1*^*tp49d*^ (received from J Malicki, University of Sheffield) and *Tg(elavl3:H2B-GcaMP6)*^*jf7Tg*^. Two photon calcium experiments were performed with larvae obtained from incrossing heterozygous *elipsa*^*+/-*^*;Tg(elavl3:H2B-GCaMP6s)* ^*jf7Tg*^ adult animals. Imaging of glutamatergic neurons were performed with larvae obtained from incrossing heterozygous *elipsa*^*+/-*^*; Tg(vglut2a:dsRed)* adult animals. For immunostaining and RNA sequencing, larvae were obtained by crossing heterozygous *elipsa*^*+/-*^ animals. Controls were either wild-type or *elipsa*^*+/-*^ obtained from the same breeding. Zebrafish larvae do not have genders.

### Method details

#### Genotyping

For genotyping, the samples were subjected to gDNA isolation using 100 μL PCR lysis buffer (containing 1M tris pH-7-9, 0.5 M EDTA, Triton-100 and Proteinase K 0.1mg/ml) overnight at 50°C. To stop the reaction the samples were heated to 95°C for 10 minutes. The samples were then centrifuged at 13000 rpm for 2 minutes. The supernatant containing gDNA was used for further KASP assays-based analysis. The samples were first diluted (1:2) with water. Further 3 μL of diluted sample was used for performing the KASP assay according to the manufacturer guidelines. The master mix contained 5 μL mastermix, 0.14 μL assay mix and 1.86 μL milliQ water per sample well.

#### Antibody staining and confocal imaging

##### Immunostaining of the brain with cilia specific antibodies

Larvae were euthanized and fixed in a solution containing 4 % paraformaldehyde solution (PFA), 1 % DMSO and 0.3 % TritonX-100 in PBS (0.3 % PBSTx) for 2 hours at room temperature or 4°C overnight. The larvae were washed with 0.3 % PBSTx after fixing to remove any traces of the fixing solution. For permeabilization, samples were incubated for 10 minutes at -20°C with acetone. Subsequently, samples were washed with 0.3 % PBSTx (3x10 min) and blocked in 0.1 % BSA made in 0.3 % PBSTx at room temperature. Samples were incubated with the primary antibody overnight at 4°C. The antibodies used were mouse glutamylated tubulin (GT335, 1:400, for staining motile cilia), mouse anti parvalbumin (1:500 for staining Purkinje cells), rabbit Arl13b antibody (1:200, for staining all cilia), or rabbit p-Histone H3 Antibody (1:500, for staining diving cells). On the second day samples were washed (0.3 % PBSTx, 3x1 hour) and subsequently incubated with the secondary antibody (Alexa-labelled GAM488 plus, or GAR555 plus Thermo Scientific, 1:1,000) and 0.1% DAPI overnight at 4°C. On the third day after incubation with the secondary antibody the larvae were washed (0.3 % PBSTx, 3x1 hour) and transferred to a series of increasing glycerol (made in PBS) concentrations (25 %, 50 % and 75 %). After staining the larvae were stored in 75 % glycerol at 4°C and imaged using a Zeiss Examiner Z1 confocal microscope with a 20x plan NA 0.8 objective. Multiple images were stitched using Fiji.^116^ For detailed protocol refer.^65^ For parvalbumin, arl13b and glutamylated tubulin staining, the larvae were dissected at 4dpf for better penetration of the antibody through the brain.

##### Immunostaining of the brain for acetylated tubulin and SV2 staining

The staining protocol for acetylated tubulin and SV2 staining was performed as per.^71^ The larvae were first euthanized and fixed in 2% trichloroacetic acid (TCA) for 3 hours at RT. After incubation, the larvae were washed 4 x 5 mins in PBS. They were then blocked with PBDT (PBS + 1% BSA + 0.5% Triton X-100 + 1% DMSO) + 10% goat serum for 30 minutes at RT. The larvae were then incubated in the primary antibodies anti acetylated tubulin (1:1000) and SV2 (1:400) diluted in PBDT + 2% serum overnight at 4°C. The next day the samples were washed through a series of washes of 10 mins, 15 mins, 30 mins and 1 hour with PBDT 6. The larvae were then incubated in secondary antibodies GAM plus (1:1000) and 0.1% dapi in PBDT at 4°C. The following day, samples were then washed with a series of washes of 10 mins, 15 mins, 30 mins and 1 hour with PDT (= PBS + 0.5% Triton X-100 + 1% DMSO). After staining the larvae were stored in 75 % glycerol at 4°C and imaged using a Zeiss Examiner Z1 confocal microscope with a 20x plan NA 0.8 objective. Multiple images were stitched using Fiji.^116^^,^^117^

#### Quantification of pH3^+^ cells, tectal neurons and measuring brain morphology

For quantifications, the images were acquired at equal height using the same z stack and the pH3^+^ cells were counted through the different planes for individual brain regions using the cell counter function in Fiji (https://imagej.nih.gov/ij/plugins/cell-counter.html). For measuring the number of tectal neurons, a defined ROI of *36x28 micron* was drawn on the z stack of the images using the rectangular tool in Fiji. The number of tectal neurons were then counted in the defined ROI (Figure 2D3) across control and mutant samples. The brain morphology measurements were done on Z stack using the straight-line tool in Fiji. The cell count data was normalized to the width of individual brain region. Normalization did not change the significance of the data, hence to avoid difficulty to interpret the data the raw counts (not normalized) have been presented in (Figure 2).

#### Cryo-sectioning and staining of the retina

The protocol for cryo-sectioning was adapted from.^118^ Zebrafish larvae were euthanized using ice water (4°C). The larvae were then fixed using 4% PFA in 0.3% triton-100 PBS overnight at 4°C. After fixing the samples were washed 3x1hour in PBS. The fish were embedded before cryoprotection, using a solution of 1.5% agarose and 5% sucrose solution in RO. The larvae were transferred to a cryomold, and the embedding solution was added to this mold. The fish were positioned using a pick and the solution was allowed to cool. After cooling, the mold was cut into tiny blocks (cut the corner to indicate orientation). The blocks were then transferred to storage solution (30% Sucrose in PBS) and stored in the fridge overnight at 4°C until the samples sank to the bottom of the tube. The next day, the blocks containing the samples were snap frozen with liquid nitrogen using a metal bowl with 2-methylbutane that is positioned in a Styrofoam container. Frozen samples were stored at -20°C until further use. Using a cryostat, sections of 10 μm thickness were cut at -30°C blade and chamber temperature. The sections were placed on super frost slides and stored at -20°C until further use. The cryosection slides were thawed at RT for 30 minutes. The slides were washed with PBS without detergent for 4x5 minutes to remove any traces of freezing medium. The outer segments were stained using DiI stain (5mg/ml diluted 1:100-1:200 in PBS) for 20 minutes at RT, followed by 3x5 minute washes with 1X PBS. The slides were then dried and mounted with prolong gold. The slides were left overnight to dry before imaging.

#### DiI-based tracing of retinal projection

4 dpf larvae were euthanized and then fixed with 4% PFA in PBS at 4 dpf O/N. Following 3 x1 hour washes with PBS, fish were immobilized with 1.5% LMP agarose in RO water. Injections into the eye were performed using an Eppendorf Femtojet 4i pressure injector and glass capillaries that were filled with 5 mg/ml DiI diluted in DMF. Following screening the larvae were imaged using a Zeiss Examiner Z1 confocal microscope with a 10x plan NA 0.45 objective.

#### RNA sequencing and transcriptomic analysis

RNA was isolated from larvae coming from four separate breedings using the same parental strain. Each batch was prepared by taking 30 larvae at 4 dpf in a 1.5ml tube. The control and the mutant tubes of each batch was processed at the same time to avoid variability. To lyse the samples, 500μL trizol was added and the euthanized larvae were homogenized through a 27-gauge needle until the mixture looked uniform. After adding another 500μL trizol, the samples were incubated for 5 minutes at room temperature. The larvae were then treated with 200μL chloroform, and the tube was rocked for 15secs to mix the contents. The tubes were incubated for 2 minutes at room temperature and then centrifuged for 15 minutes at 12000rpm at a temperature of 4°C. After centrifugation, the upper aqueous phase containing RNA was mixed with equal amounts of 100% ethanol and was then loaded onto an RNA spin column (Qiagen) and centrifuged for 30 seconds at 8000 rpm. The spin column was further incubated with 700μL of RW1 buffer and centrifuged for 30 seconds at 8000 rpm. The spin column tubes were then placed into a new collection tube and further treated to remove any DNA contamination by washing the tubes with 350μL of RW1 buffer followed by Dnase enzyme (Qiagen) in RDD buffer (10μL Dnase+ 70μL RDD buffer per tube) for 45 minutes at room temperature. After incubation, 350μL of RW1 buffer was added to the tubes and centrifuged for 15 seconds at 8000rpm. The tubes were then treated with 500μL RPE buffer and centrifuged for 30 seconds. This step was repeated twice, and the tubes were then centrifuged for 1 minute at 8000rpm to remove any residual buffer left in the column. For RNA extraction from the column, 30μL nuclease free water was added and incubated for 2 minutes. The tubes were then centrifuged for 1 minute at 8000 rpm to elute the RNA. The concentration of the extracted RNA was quantified using Nanodrop and the quality was analyzed by bioanalyzer. The samples were then sequenced using BGI’s DNBSEQTM Technology using the Dr Tom data visualization and analysis platform provided by BGI. The filtering of sequencing data was done using SOAPnuke, v1.5.2, using the parameters Parameters -l 15 -q 0.2 -n 0.05. The Hierarchical Indexing for Spliced Alignment of Transcripts software (HISAT2 v2.0.4 with parameters:--sensitive --no-discordant --no-mixed -I 1 -X 1000 -p 8 --rna-strandness RF) was used for mapping RNA-seq reads. We used Bowtie2^119^ (Version:v2.2.5, Parameters:-q --sensitive --dpad 0 --gbar 99999999 --mp 1,1 --np 1 --score-min L,0,-0.1 -p 16 -k 200) to map the clean reads to the reference gene sequence (transcriptome), and then RSEM^120^(Version:v1.2.8, Parameters:-p 8 --forward-prob 0 --paired-end) to calculate the gene expression level of each sample. The count matrix included genes that were selected based on their expression of average count per million of more than 1 across all samples. The resulting matrix was normalized, and log transformed using the voom algorithm from the limma package of Bioconductor.^121^^,^^122^ The heatmap (Figure 4A1) was generated in MATLAB using the inbuilt clustergram function (clustergram(matrix,'Standardize','row','Colormap',cmap)).

For performing gene ontology (GO) analysis, the differentially expressed genes having a p-adjusted value lower than 0.1 were selected and analyzed using the DAVID tool^123^^,^^124^ https://david.ncifcrf.gov/home.jsp. GOTERM_BP_FAT, GOTERM_CC_FAT, and GOTERM_MF_FAT were used with a count threshold of 5 and a EASE threshold of 0.05. We selected the top 20 GO term based on the FDR. We used as background all genes that were detected in our RNA sequencing. The output files, containing the associated genes ID, were downloaded, and processed to generate the heatmaps shown in Figures 4D1–4D4 that represent z-score data (log2 normalized read counts) for each gene across the 8 samples (4 controls and 4 mutants).

Raw data are deposited on the GEO repository with the accession number GSE244171.

#### Two photon calcium imaging

Two- photon calcium imaging was performed on 4 dpf *elipsa;Tg(elavl3:H2B-GCaMP6)* ^*jf7Tg*^. The larvae were paralyzed upon injection of α-bungarotoxin (Invitrogen BI601, 1 mg/ml) and embedded in 1.5% low melting point agarose in mounting chambers (Fluorodish, World Precision Instruments) using a plastic microcapillary tip, as described in.^44^ After 10 min of agarose solidification, 750 μL of AFW was added on top of the agarose. The mounting chamber was then allowed to stabilize under the two-photon microscope before recording. The recordings were performed in a two-photon microscope (Scientifica) using a 16× water immersion objective (Nikon, numerical aperture 0.8, Long Working Distance 3.0, plan) and a Ti:Sapphire laser (MaiTai Spectra-Physics) tuned at 920 nm. Recordings of 1536 ×512 pixels and 8 planes were acquired at a frame rate of 30.85 frames per second and a volume rate of 3.86 Hz. The total recording time was 40 minutes (74000 frames). Ongoing activity was recorded for 10 minutes in darkness, followed by 5 stimuli each of 60 seconds using a red light-emitting diode light (LZ1-00R105, LedEngin; 625 nm), at minute 10, 15, 20, 25 and 30 respectively. Animals without cerebral blood flow after the experiments were excluded from the analysis.

#### Data analysis

Two-photon microscopy images were aligned using a previously reported algorithm.^44^^,^^79^ The recordings were then screened manually to check for movement and Z drift. Unstable recordings were discarded from the analysis. All analyses were performed on MATLAB. Neurons were segmented using a pattern recognition algorithm adapted from^125^ with torus or ring shaped neuronal templates.^106^ The identified neurons were then set apart into different brain regions based on another algorithm described in^80^ (Figure 6A2). Clustering of neurons was performed using k-means clustering algorithm based on their activity.^106^ For detecting light responses, we used the 5 seconds of fluorescence preceding the stimuli as baseline and average of the 5 stimuli for each neuron. The 5s baseline was used to calculate dF/F. Responsive cells were selected based on their response during the 10 seconds following the light onset or offset. Cells were classified as responsive if their average activity during the 10s window was greater than 2 ∗ standard deviation of the baseline. Neurons with an average activity below 2 ∗ standard deviation of the baseline were considered as inhibited.

For measuring spontaneous activity, we excluded the first two minutes of the recordings (to avoid artefacts from the microscope laser turning on). We selected frames 460-2304 (a total of 7.5 minutes of ongoing activity). dF/F was calculated using, as baseline, the 8^th^ percentile of a moving window as previously described.^79^^,^^126^ The data was resampled to 1fps using the resample function in MATLAB. Calculations for the activity of cells were done on resampled data. A cell was considered active at a given second if it had an activity higher than a threshold of 4∗ 8^th^ percentile. We then calculated the percentage of cells that are very active (active more than 50% of the time) or inactive (active less than 10% of the time) per brain region. The Pearson’s correlation versus distance between neurons was calculated up to 60μm per brain region (Figure S5).

#### Electroretinography

Electroretinography (ERG) were performed in control or *elipsa* mutants at 4dpf. First, we selected healthy control or *elipsa* mutants at 4dpf and anesthetized the larvae by using MS222. We then placed the anesthetized larvae on a wet filter paper (VWR, 516-0848) on a FluoroDish (VWR, FD35PDL-100) and covered their trunk with paper towels to affix them. We used a sliver-coated wire as a reference electrode, which was chlorinated by immersing in sodium hypochlorite solution 10 minutes before the recordings. The reference electrode was positioned on the same bath as the larvae. Recording electrodes were pulled from glass capillaries (WPI, TW100F-4) to have a 15-30 μm opening and filled with artificial fish water (AFW) containing MS222. The electrode was placed on the cornea of the eye using a motorized micromanipulator (Scientifica). Before the recording, larvae were subjected to over 10 minutes dark adaption. The voltages were measured by a MultiClamp 700B device (Molecular Devices) with a 2kHz low-pass filter and were digitized at 10 kHz. A train of 10 light stimuli was initiated at the 5^th^ second of the recording. The stimuli were produced by a blue LED using a pulse generator (Master-8, AMPI). Each stimulus (light intensity: 0.005-0.007 mW) lasted 1 sec with 5 seconds intervals between stimuli. The total time for each recording was 60 seconds. We performed minimum 5 recordings for each larva and selected the trial that shows the most representative responses for further analysis. We utilized custom MATLAB scripts for data acquisition and all subsequent analyses. To quantify the voltage responses, we used a 200 msec baseline before the light stimulus to normalize the traces and averaged the 10 light stimuli. We calculated the average voltage responses for all control and mutants from 0 to 200 msecs after the light ON stimuli, which show the strong b-wave representing the ON bipolar cell response.

### Quantification and statistical analysis

Statistical analysis was done using MATLAB. Wilcoxon rank-sum test was used for nonpaired analysis. Probability of *p* < 0.05 was considered statistically significant.

## Supplemental information

Document S1. Figures S1–S5

Table S1. Differentially expressed genes and normalized counts obtained by RNA sequencing, related to Figure 4 Fold change, p-values and log2-transformed normalized counts obtained from 4 dpf control (CTRL1-4) and *elipsa* mutant (MUT1-4) larvae. 4 independent breeding and RNA isolation were performed on control and mutant siblings. Differentially expressed genes based on a threshold of p-value adjusted of 0.1 are highlighted in yellow.

Table S2. Enriched GO terms, related to Figure 4 Output of GO analysis from DAVID with enriched GO terms and their associated differentially expressed genes.

## References

[1] Mitchison H.M., Valente E.M. (2017). Motile and non-motile cilia in human pathology: from function to phenotypes. J. Pathol..

[2] Nachury M.V. (2014). How do cilia organize signalling cascades?. Philos. Trans. R. Soc. Lond. B Biol. Sci..

[3] Louvi A., Grove E.A. (2011). Cilia in the CNS: The Quiet Organelle Claims Center Stage. Neuron.

[4] Gopalakrishnan J., Feistel K., Friedrich B.M., Grapin-Botton A., Jurisch-Yaksi N., Mass E., Mick D.U., Müller R.U., May-Simera H., Schermer B. (2023). Emerging principles of primary cilia dynamics in controlling tissue organization and function. EMBO J..

[5] Guemez-Gamboa A., Coufal N.G., Gleeson J.G. (2014). Primary cilia in the developing and mature brain. Neuron.

[6] Ringers C., Olstad E.W., Jurisch-Yaksi N. (2020). The role of motile cilia in the development and physiology of the nervous system. Philos. Trans. R. Soc. Lond. B Biol. Sci..

[7] Bear R.M., Caspary T. (2024). Uncovering cilia function in glial development. Ann. Hum. Genet..

[8] Falk N., Lösl M., Schröder N., Gießl A. (2015). Specialized Cilia in Mammalian Sensory Systems. Cells.

[9] Bachmann-Gagescu R., Neuhauss S.C. (2019). The photoreceptor cilium and its diseases. Curr. Opin. Genet. Dev..

[10] Suciu S.K., Caspary T. (2021). Cilia, neural development and disease. Semin. Cell Dev. Biol..

[11] Jurisch-Yaksi N., Wachten D., Gopalakrishnan J. (2024). The neuronal cilium - a highly diverse and dynamic organelle involved in sensory detection and neuromodulation. Trends Neurosci..

[12] Hansen J.N., Rassmann S., Stüven B., Jurisch-Yaksi N., Wachten D. (2021). CiliaQ: a simple, open-source software for automated quantification of ciliary morphology and fluorescence in 2D, 3D, and 4D images. Eur. Phys. J. E Soft Matter.

[13] Paridaen J.T.M.L., Huttner W.B. (2014). Neurogenesis during development of the vertebrate central nervous system. EMBO Rep..

[14] Gabriel E., Wason A., Ramani A., Gooi L.M., Keller P., Pozniakovsky A., Poser I., Noack F., Telugu N.S., Calegari F. (2016). CPAP promotes timely cilium disassembly to maintain neural progenitor pool. EMBO J..

[15] Wachten D., Mick D.U. (2021). Signal transduction in primary cilia – analyzing and manipulating GPCR and second messenger signaling. Pharmacol. Ther..

[16] Hilgendorf K.I., Myers B.R., Reiter J.F. (2024). Emerging mechanistic understanding of cilia function in cellular signalling. Nat. Rev. Mol. Cell Biol..

[17] Bangs F., Anderson K.V. (2017). Primary cilia and mammalian hedgehog signaling. Cold Spring Harb. Perspect. Biol..

[18] Huangfu D., Liu A., Rakeman A.S., Murcia N.S., Niswander L., Anderson K.V. (2003). Hedgehog signalling in the mouse requires intraflagellar transport proteins. Nature.

[19] Youn Y.H., Han Y.G. (2018). Primary Cilia in Brain Development and Diseases. Am. J. Pathol..

[20] Higginbotham H., Guo J., Yokota Y., Umberger N.L., Su C.Y., Li J., Verma N., Hirt J., Ghukasyan V., Caspary T., Anton E.S. (2013). Arl13b-regulated cilia activities are essential for polarized radial glial scaffold formation. Nat. Neurosci..

[21] Stoufflet J., Caillé I. (2022). The Primary Cilium and Neuronal Migration. Cells.

[22] Higginbotham H., Eom T.Y., Mariani L.E., Bachleda A., Hirt J., Gukassyan V., Cusack C.L., Lai C., Caspary T., Anton E.S. (2012). Arl13b in primary cilia regulates the migration and placement of interneurons in the developing cerebral cortex. Dev. Cell.

[23] Stoufflet J., Chaulet M., Doulazmi M., Fouquet C., Dubacq C., Métin C., Schneider-Maunoury S., Trembleau A., Vincent P., Caillé I. (2020). Primary cilium-dependent cAMP/PKA signaling at the centrosome regulates neuronal migration. Sci. Adv..

[24] Guo J., Otis J.M., Suciu S.K., Catalano C., Xing L., Constable S., Wachten D., Gupton S., Lee J., Lee A. (2019). Primary Cilia Signaling Promotes Axonal Tract Development and Is Disrupted in Joubert Syndrome-Related Disorders Models. Dev. Cell.

[25] Kumamoto N., Gu Y., Wang J., Janoschka S., Takemaru K.I., Levine J., Ge S. (2012). A role for primary cilia in glutamatergic synaptic integration of adult-born neurons. Nat. Neurosci..

[26] Guo J., Otis J.M., Higginbotham H., Monckton C., Cheng J., Asokan A., Mykytyn K., Caspary T., Stuber G.D., Anton E.S. (2017). Primary Cilia Signaling Shapes the Development of Interneuronal Connectivity. Dev. Cell.

[27] Guo J., Higginbotham H., Li J., Nichols J., Hirt J., Ghukasyan V., Anton E.S. (2015). Developmental disruptions underlying brain abnormalities in ciliopathies. Nat. Commun..

[28] McClintock T.S., Khan N., Xie C., Martens J.R. (2020). Maturation of the Olfactory Sensory Neuron and Its Cilia. Chem. Senses.

[29] Bergboer J.G.M., Wyatt C., Austin-Tse C., Yaksi E., Drummond I.A. (2018). Assaying sensory ciliopathies using calcium biosensor expression in zebrafish ciliated olfactory neurons. Cilia.

[30] Rayamajhi D., Ege M., Ukhanov K., Ringers C., Zhang Y., Jung I., D'Gama P.P., Li S.S., Cosacak M.I., Kizil C. (2024). The forkhead transcription factor Foxj1 controls vertebrate olfactory cilia biogenesis and sensory neuron differentiation. PLoS Biol..

[31] Insinna C., Besharse J.C. (2008). Intraflagellar transport and the sensory outer segment of vertebrate photoreceptors. Dev. Dyn..

[32] Bujakowska K.M., Liu Q., Pierce E.A. (2017). Photoreceptor Cilia and Retinal Ciliopathies. Cold Spring Harb. Perspect. Biol..

[33] DeMars K.M., Ross M.R., Starr A., McIntyre J.C. (2023). Neuronal primary cilia integrate peripheral signals with metabolic drives. Front. Physiol..

[34] Sheu S.-H., Upadhyayula S., Dupuy V., Pang S., Deng F., Wan J., Walpita D., Pasolli H.A., Houser J., Sanchez-Martinez S. (2022). A serotonergic axon-cilium synapse drives nuclear signaling to alter chromatin accessibility. Cell.

[35] Wang Y., Bernard A., Comblain F., Yue X., Paillart C., Zhang S., Reiter J.F., Vaisse C. (2021). Melanocortin 4 receptor signals at the neuronal primary cilium to control food intake and body weight. J. Clin. Invest..

[36] Tu H.-Q., Li S., Xu Y.-L., Zhang Y.-C., Li P.-Y., Liang L.-Y., Song G.-P., Jian X.-X., Wu M., Song Z.-Q. (2023). Rhythmic cilia changes support SCN neuron coherence in circadian clock. Science.

[37] Berbari N.F., Lewis J.S., Bishop G.A., Askwith C.C., Mykytyn K. (2008). Bardet-Biedl syndrome proteins are required for the localization of G protein-coupled receptors to primary cilia. Proc. Natl. Acad. Sci. USA.

[38] Händel M., Schulz S., Stanarius A., Schreff M., Erdtmann-Vourliotis M., Schmidt H., Wolf G., Höllt V. (1999). Selective targeting of somatostatin receptor 3 to neuronal cilia. Neuroscience.

[39] Hilgendorf K.I., Johnson C.T., Jackson P.K. (2016). The primary cilium as a cellular receiver: organizing ciliary GPCR signaling. Curr. Opin. Cell Biol..

[40] Hamon M., Doucet E., Lefèvre K., Miquel M.-C., Lanfumey L., Insausti R., Frechilla D., Del Rio J., Vergé D. (1999). Antibodies and Antisense Oligonucleotide for Probing the Distribution and Putative Functions of Central 5-HT6 Receptors. Neuropsychopharmacology.

[41] Domire J.S., Green J.A., Lee K.G., Johnson A.D., Askwith C.C., Mykytyn K. (2011). Dopamine receptor 1 localizes to neuronal cilia in a dynamic process that requires the Bardet-Biedl syndrome proteins. Cell. Mol. Life Sci..

[42] Menco B.P., Cunningham A.M., Qasba P., Levy N., Reed R.R. (1997). Putative odour receptors localize in cilia of olfactory receptor cells in rat and mouse: a freeze-substitution ultrastructural study. J. Neurocytol..

[43] Sengupta P., Chou J.H., Bargmann C.I. (1996). odr-10 Encodes a Seven Transmembrane Domain Olfactory Receptor Required for Responses to the Odorant Diacetyl. Cell.

[44] Reiten I., Uslu F.E., Fore S., Pelgrims R., Ringers C., Diaz Verdugo C., Hoffman M., Lal P., Kawakami K., Pekkan K. (2017). Motile-Cilia-Mediated Flow Improves Sensitivity and Temporal Resolution of Olfactory Computations. Curr. Biol..

[45] Fliegauf M., Benzing T., Omran H. (2007). When cilia go bad: cilia defects and ciliopathies. Nat. Rev. Mol. Cell Biol..

[46] Del Bigio M.R. (2010). Ependymal cells: biology and pathology. Acta Neuropathol..

[47] Faubel R., Westendorf C., Bodenschatz E., Eichele G. (2016). Cilia-based flow network in the brain ventricles. Science.

[48] Olstad E.W., Ringers C., Hansen J.N., Wens A., Brandt C., Wachten D., Yaksi E., Jurisch-Yaksi N. (2019). Ciliary Beating Compartmentalizes Cerebrospinal Fluid Flow in the Brain and Regulates Ventricular Development. Curr. Biol..

[49] Sawamoto K., Wichterle H., Gonzalez-Perez O., Cholfin J.A., Yamada M., Spassky N., Murcia N.S., Garcia-Verdugo J.M., Marin O., Rubenstein J.L.R. (2006). New neurons follow the flow of cerebrospinal fluid in the adult brain. Science.

[50] D'Gama P.P., Qiu T., Cosacak M.I., Rayamajhi D., Konac A., Hansen J.N., Ringers C., Acuña-Hinrichsen F., Hui S.P., Olstad E.W. (2021). Diversity and function of motile ciliated cell types within ependymal lineages of the zebrafish brain. Cell Rep..

[51] D’Gama P.P., Jeong I., Nygård A.M., Jamali A., Yaksi E., Jurisch-Yaksi N. (2024). Cilia-mediated cerebrospinal fluid flow modulates neuronal and astroglial activity in the zebrafish larval brain. bioRxiv.

[52] Reiter J.F., Leroux M.R. (2017). Genes and molecular pathways underpinning ciliopathies. Nat. Rev. Mol. Cell Biol..

[53] Andreu-Cervera A., Catala M., Schneider-Maunoury S. (2021). Cilia, ciliopathies and hedgehog-related forebrain developmental disorders. Neurobiol. Dis..

[54] Forsythe E., Beales P.L. (2013). Bardet–Biedl syndrome. Eur. J. Hum. Genet..

[55] Forsyth R., Gunay-Aygun M., Adam M.P., Mirzaa G.M., Pagon R.A., Wallace S.E., Bean L.J.H., Gripp K.W., Amemiya A. (1993). GeneReviews(®).

[56] Bachmann-Gagescu R., Dempsey J.C., Bulgheroni S., Chen M.L., D'Arrigo S., Glass I.A., Heller T., Héon E., Hildebrandt F., Joshi N. (2020). Healthcare recommendations for Joubert syndrome. Am. J. Med. Genet..

[57] Joubert M., Eisenring J.J., Robb J.P., Andermann F. (1969). Familial agenesis of the cerebellar vermis. A syndrome of episodic hyperpnea, abnormal eye movements, ataxia, and retardation. Neurology.

[58] Parisi M., Glass I., Adam M.P., Mirzaa G.M., Pagon R.A., Wallace S.E., Bean L.J.H., Gripp K.W., Amemiya A. (1993). GeneReviews(®).

[59] Brancati F., Dallapiccola B., Valente E.M. (2010). Joubert Syndrome and related disorders. Orphanet J. Rare Dis..

[60] Ferland R.J., Eyaid W., Collura R.V., Tully L.D., Hill R.S., Al-Nouri D., Al-Rumayyan A., Topcu M., Gascon G., Bodell A. (2004). Abnormal cerebellar development and axonal decussation due to mutations in AHI1 in Joubert syndrome. Nat. Genet..

[61] Bachmann-Gagescu R., Dempsey J.C., Phelps I.G., O'Roak B.J., Knutzen D.M., Rue T.C., Ishak G.E., Isabella C.R., Gorden N., Adkins J. (2015). Joubert syndrome: a model for untangling recessive disorders with extreme genetic heterogeneity. J. Med. Genet..

[62] Omori Y., Zhao C., Saras A., Mukhopadhyay S., Kim W., Furukawa T., Sengupta P., Veraksa A., Malicki J. (2008). Elipsa is an early determinant of ciliogenesis that links the IFT particle to membrane-associated small GTPase Rab8. Nat. Cell Biol..

[63] Bahadori R., Huber M., Rinner O., Seeliger M.W., Geiger-Rudolph S., Geisler R., Neuhauss S.C.F. (2003). Retinal function and morphology in two zebrafish models of oculo-renal syndromes. Eur. J. Neurosci..

[64] Duldulao N.A., Lee S., Sun Z. (2009). Cilia localization is essential for *in vivo* functions of the Joubert syndrome protein Arl13b/Scorpion. Development.

[65] D'Gama P.P., Jurisch-Yaksi N. (2023). Methods in Cell Biology.

[66] Prigent C., Dimitrov S. (2003). Phosphorylation of serine 10 in histone H3, what for?. J. Cell Sci..

[67] Sattar S., Gleeson J.G. (2011). The ciliopathies in neuronal development: a clinical approach to investigation of Joubert syndrome and Joubert syndrome-related disorders. Dev. Med. Child Neurol..

[68] Spassky N., Han Y.G., Aguilar A., Strehl L., Besse L., Laclef C., Ros M.R., Garcia-Verdugo J.M., Alvarez-Buylla A. (2008). Primary cilia are required for cerebellar development and Shh-dependent expansion of progenitor pool. Dev. Biol..

[69] Leto K., Carletti B., Williams I.M., Magrassi L., Rossi F. (2006). Different types of cerebellar GABAergic interneurons originate from a common pool of multipotent progenitor cells. J. Neurosci..

[70] Bae Y.-K., Kani S., Shimizu T., Tanabe K., Nojima H., Kimura Y., Higashijima S.-I., Hibi M. (2009). Anatomy of zebrafish cerebellum and screen for mutations affecting its development. Dev. Biol..

[71] Noble A.R., Masek M., Hofmann C., Cuoco A., Rusterholz T.D.S., Özkoc H., Greter N.R., Vladimirov N., Kollmorgen S., Stoeckli E., Bachmann-Gagescu R. (2024). Shared and unique consequences of Joubert Syndrome gene dysfunction on the zebrafish central nervous system. bioRxiv.

[72] Miyasaka N., Morimoto K., Tsubokawa T., Higashijima S.i., Okamoto H., Yoshihara Y. (2009). From the olfactory bulb to higher brain centers: genetic visualization of secondary olfactory pathways in zebrafish. J. Neurosci..

[73] Choksi S.P., Babu D., Lau D., Yu X., Roy S. (2014). Systematic discovery of novel ciliary genes through functional genomics in the zebrafish. Development.

[74] Djebar M., Anselme I., Pezeron G., Bardet P.L., Cantaut-Belarif Y., Eschstruth A., López Santos D., Hélène L.R., Jenett A., Khoury H. (2024). Astrogliosis and Neuroinflammation Underlie Scoliosis Upon Cilia Dysfunction. bioRxiv.

[75] Cacialli P., Gueguen M.M., Coumailleau P., D'Angelo L., Kah O., Lucini C., Pellegrini E. (2016). BDNF Expression in Larval and Adult Zebrafish Brain: Distribution and Cell Identification. PLoS One.

[76] Vladimirov N., Mu Y., Kawashima T., Bennett D.V., Yang C.T., Looger L.L., Keller P.J., Freeman J., Ahrens M.B. (2014). Light-sheet functional imaging in fictively behaving zebrafish. Nat. Methods.

[77] Myren-Svelstad S., Jamali A., Ophus S.S., D'gama P.P., Ostenrath A.M., Mutlu A.K., Hoffshagen H.H., Hotz A.L., Neuhauss S.C.F., Jurisch-Yaksi N., Yaksi E. (2022). Elevated photic response is followed by a rapid decay and depressed state in ictogenic networks. Epilepsia.

[78] Stringer C., Zhong L., Syeda A., Du F., Kesa M., Pachitariu M. (2023). Rastermap: a discovery method for neural population recordings. bioRxiv.

[79] Fore S., Acuña-Hinrichsen F., Mutlu K.A., Bartoszek E.M., Serneels B., Faturos N.G., Chau K.T.P., Cosacak M.I., Verdugo C.D., Palumbo F. (2020). Functional properties of habenular neurons are determined by developmental stage and sequential neurogenesis. Sci. Adv..

[80] Bartoszek E.M., Ostenrath A.M., Jetti S.K., Serneels B., Mutlu A.K., Chau K.T.P., Yaksi E. (2021). Ongoing habenular activity is driven by forebrain networks and modulated by olfactory stimuli. Curr. Biol..

[81] Diaz Verdugo C., Myren-Svelstad S., Aydin E., Van Hoeymissen E., Deneubourg C., Vanderhaeghe S., Vancraeynest J., Pelgrims R., Cosacak M.I., Muto A. (2019). Glia-neuron interactions underlie state transitions to generalized seizures. Nat. Commun..

[82] Doerre G., Malicki J. (2002). Genetic analysis of photoreceptor cell development in the zebrafish retina. Mech. Dev..

[83] Cantaut-Belarif Y., Sternberg J.R., Thouvenin O., Wyart C., Bardet P.L. (2018). The Reissner Fiber in the Cerebrospinal Fluid Controls Morphogenesis of the Body Axis. Curr. Biol..

[84] Liu S., Trupiano M.X., Simon J., Guo J., Anton E.S. (2021). The essential role of primary cilia in cerebral cortical development and disorders. Curr. Top. Dev. Biol..

[85] Park S.M., Jang H.J., Lee J.H. (2019). Roles of Primary Cilia in the Developing Brain. Front. Cell. Neurosci..

[86] Rusterholz T.D.S., Hofmann C., Bachmann-Gagescu R. (2022). Insights Gained From Zebrafish Models for the Ciliopathy Joubert Syndrome. Front. Genet..

[87] Bashford A.L., Subramanian V. (2019). Mice with a conditional deletion of Talpid3 (KIAA0586) - a model for Joubert syndrome. J. Pathol..

[88] Rachel R.A., Yamamoto E.A., Dewanjee M.K., May-Simera H.L., Sergeev Y.V., Hackett A.N., Pohida K., Munasinghe J., Gotoh N., Wickstead B. (2015). CEP290 alleles in mice disrupt tissue-specific cilia biogenesis and recapitulate features of syndromic ciliopathies. Hum. Mol. Genet..

[89] Damerla R.R., Cui C., Gabriel G.C., Liu X., Craige B., Gibbs B.C., Francis R., Li Y., Chatterjee B., San Agustin J.T. (2015). Novel Jbts17 mutant mouse model of Joubert syndrome with cilia transition zone defects and cerebellar and other ciliopathy related anomalies. Hum. Mol. Genet..

[90] Zhu J., Wang H.T., Chen Y.R., Yan L.Y., Han Y.Y., Liu L.Y., Cao Y., Liu Z.Z., Xu H.A. (2020). The Joubert Syndrome Gene arl13b is Critical for Early Cerebellar Development in Zebrafish. Neurosci. Bull..

[91] Plotnikova O.V., Pugacheva E.N., Golemis E.A. (2009). Primary cilia and the cell cycle. Methods Cell Biol..

[92] Tucker R.W., Pardee A.B., Fujiwara K. (1979). Centriole ciliation is related to quiescence and DNA synthesis in 3T3 cells. Cell.

[93] Jurisch-Yaksi N., Yaksi E., Kizil C. (2020). Radial glia in the zebrafish brain: Functional, structural, and physiological comparison with the mammalian glia. Glia.

[94] Lindsey B.W., Hall Z.J., Heuzé A., Joly J.-S., Tropepe V., Kaslin J. (2018). The role of neuro-epithelial-like and radial-glial stem and progenitor cells in development, plasticity, and repair. Prog. Neurobiol..

[95] Recher G., Jouralet J., Brombin A., Heuzé A., Mugniery E., Hermel J.-M., Desnoulez S., Savy T., Herbomel P., Bourrat F. (2013). Zebrafish midbrain slow-amplifying progenitors exhibit high levels of transcripts for nucleotide and ribosome biogenesis. Development.

[96] Robles E., Laurell E., Baier H. (2014). The Retinal Projectome Reveals Brain-Area-Specific Visual Representations Generated by Ganglion Cell Diversity. Curr. Biol..

[97] Mueller T. (2012). What is the Thalamus in Zebrafish?. Front. Neurosci..

[98] Dreosti E., Vendrell Llopis N., Carl M., Yaksi E., Wilson S.W. (2014). Left-Right Asymmetry Is Required for the Habenulae to Respond to Both Visual and Olfactory Stimuli. Curr. Biol..

[99] Zhang B.-B., Yao Y.-Y., Zhang H.-F., Kawakami K., Du J.-L. (2017). Left Habenula Mediates Light-Preference Behavior in Zebrafish via an Asymmetrical Visual Pathway. Neuron.

[100] Vigh B., Manzano M.J., Zádori A., Frank C.L., Lukáts A., Röhlich P., Szél A., Dávid C. (2002). Nonvisual photoreceptors of the deep brain, pineal organs and retina. Histol. Histopathol..

[101] Laurà R., Magnoli D., Zichichi R., Guerrera M.C., De Carlos F., Suárez A.Á., Abbate F., Ciriaco E., Vega J.A., Germanà A. (2012). The photoreceptive cells of the pineal gland in adult zebrafish (Danio rerio). Microsc. Res. Tech..

[102] Fernandes A.M., Fero K., Arrenberg A.B., Bergeron S.A., Driever W., Burgess H.A. (2012). Deep Brain Photoreceptors Control Light-Seeking Behavior in Zebrafish Larvae. Curr. Biol..

[103] Dekens M.P.S., Fontinha B.M., Gallach M., Pflügler S., Tessmar-Raible K. (2022). Melanopsin elevates locomotor activity during the wake state of the diurnal zebrafish. EMBO Rep..

[104] Fontinha B.M., Zekoll T., Al-Rawi M., Gallach M., Reithofer F., Barker A.J., Hofbauer M., Fischer R.M., von Haeseler A., Baier H., Tessmar-Raible K. (2021). TMT-Opsins differentially modulate medaka brain function in a context-dependent manner. PLoS Biol..

[105] Lepanto P., Davison C., Casanova G., Badano J.L., Zolessi F.R. (2016). Characterization of primary cilia during the differentiation of retinal ganglion cells in the zebrafish. Neural Dev..

[106] Jetti S.K., Vendrell-Llopis N., Yaksi E. (2014). Spontaneous activity governs olfactory representations in spatially organized habenular microcircuits. Curr. Biol..

[107] Kirkby L.A., Sack G.S., Firl A., Feller M.B. (2013). A Role for Correlated Spontaneous Activity in the Assembly of Neural Circuits. Neuron.

[108] Choi B.J., Chen Y.C.D., Desplan C. (2021). Building a circuit through correlated spontaneous neuronal activity in the developing vertebrate and invertebrate visual systems. Genes Dev..

[109] Uddin L.Q. (2020). Bring the Noise: Reconceptualizing Spontaneous Neural Activity. Trends Cogn. Sci..

[110] Liu Y., Nour M.M., Schuck N.W., Behrens T.E.J., Dolan R.J. (2022). Decoding cognition from spontaneous neural activity. Nat. Rev. Neurosci..

[111] Hotz A.L., Jamali A., Rieser N.N., Niklaus S., Aydin E., Myren-Svelstad S., Lalla L., Jurisch-Yaksi N., Yaksi E., Neuhauss S.C.F. (2022). Loss of glutamate transporter eaat2a leads to aberrant neuronal excitability, recurrent epileptic seizures, and basal hypoactivity. Glia.

[112] Yaksi E., Jamali A., Diaz Verdugo C., Jurisch-Yaksi N. (2021). Past, present and future of zebrafish in epilepsy research. FEBS J..

[113] Samarut É., Swaminathan A., Riché R., Liao M., Hassan-Abdi R., Renault S., Allard M., Dufour L., Cossette P., Soussi-Yanicostas N., Drapeau P. (2018). γ-Aminobutyric acid receptor alpha 1 subunit loss of function causes genetic generalized epilepsy by impairing inhibitory network neurodevelopment. Epilepsia.

[114] Niklaus S., Cadetti L., Vom Berg-Maurer C.M., Lehnherr A., Hotz A.L., Forster I.C., Gesemann M., Neuhauss S.C.F. (2017). Shaping of Signal Transmission at the Photoreceptor Synapse by EAAT2 Glutamate Transporters. eNeuro.

[115] Han S., Miyoshi K., Shikada S., Amano G., Wang Y., Yoshimura T., Katayama T. (2019). TULP3 is required for localization of membrane-associated proteins ARL13B and INPP5E to primary cilia. Biochem. Biophys. Res. Commun..

[116] Preibisch S., Saalfeld S., Tomancak P. (2009). Globally optimal stitching of tiled 3D microscopic image acquisitions. Bioinformatics.

[117] Schindelin J., Arganda-Carreras I., Frise E., Kaynig V., Longair M., Pietzsch T., Preibisch S., Rueden C., Saalfeld S., Schmid B. (2012). Fiji: an open-source platform for biological-image analysis. Nat. Methods.

[118] Masek M., Zang J., Mateos J.M., Garbelli M., Ziegler U., Neuhauss S.C.F., Bachmann-Gagescu R. (2023). Studying the morphology, composition and function of the photoreceptor primary cilium in zebrafish. Methods Cell Biol..

[119] Langmead B., Salzberg S.L. (2012). Fast gapped-read alignment with Bowtie 2. Nat. Methods.

[120] Li B., Dewey C.N. (2011). RSEM: accurate transcript quantification from RNA-Seq data with or without a reference genome. BMC Bioinf..

[121] Ritchie M.E., Phipson B., Wu D., Hu Y., Law C.W., Shi W., Smyth G.K. (2015). limma powers differential expression analyses for RNA-sequencing and microarray studies. Nucleic Acids Res..

[122] Law C.W., Chen Y., Shi W., Smyth G.K. (2014). voom: Precision weights unlock linear model analysis tools for RNA-seq read counts. Genome Biol..

[123] Huang D.W., Sherman B.T., Lempicki R.A. (2009). Systematic and integrative analysis of large gene lists using DAVID bioinformatics resources. Nat. Protoc..

[124] Sherman B.T., Hao M., Qiu J., Jiao X., Baseler M.W., Lane H.C., Imamichi T., Chang W. (2022). DAVID: a web server for functional enrichment analysis and functional annotation of gene lists (2021 update). Nucleic Acids Res..

[125] Ohki K., Chung S., Ch'ng Y.H., Kara P., Reid R.C. (2005). Functional imaging with cellular resolution reveals precise micro-architecture in visual cortex. Nature.

[126] Romano S.A., Pérez-Schuster V., Jouary A., Boulanger-Weill J., Candeo A., Pietri T., Sumbre G. (2017). An integrated calcium imaging processing toolbox for the analysis of neuronal population dynamics. PLoS Comput. Biol..

