# Supplementary material for: Ciliogenesis defects after neurulation impact brain development and neuronal activity in larval zebrafish
Source: iScience. 2024 May 22;27(6):110078. doi: 10.1016/j.isci.2024.110078 (PMC11167523; doi:10.1016/j.isci.2024.110078)
Supplement: Document S1. Figures S1–S5 [file mmc1.pdf]

iScience, Volume *27*

Supplemental information

Ciliogenesis defects after neurulation impact brain development and neuronal activity in larval zebrafish

Percival P. D'Gama, Inyoung Jeong, Andreas Moe Nygård, Anh-Tuan Trinh, Emre Yaksi, and Nathalie Jurisch-Yaksi

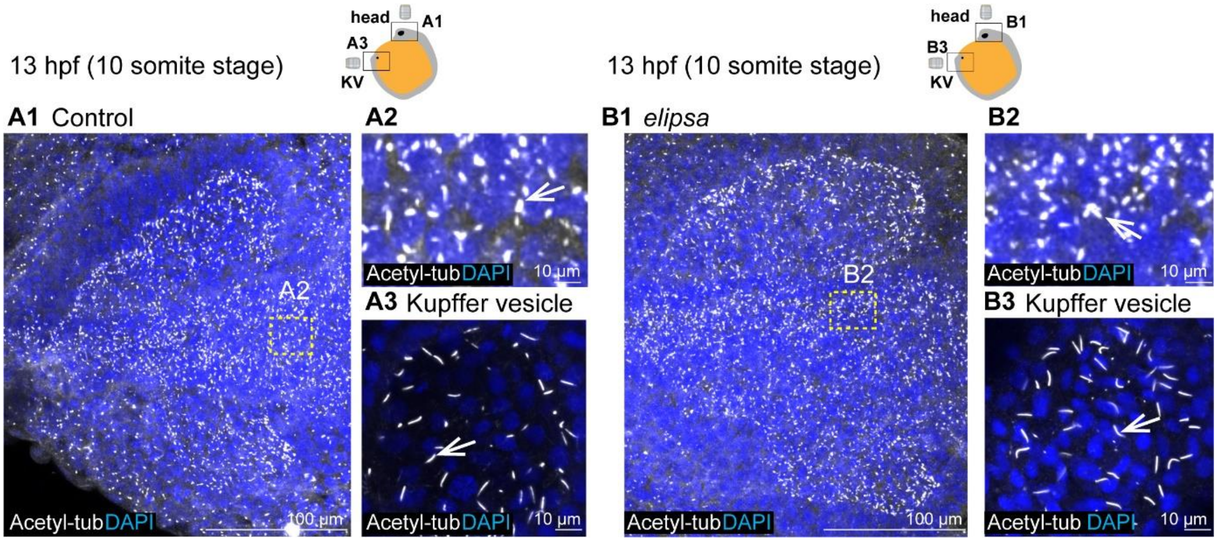

Supplemental figure S1: Cilia are normal at 10 somites stage in *elipsa* mutants. Related to figure 1 **(A1-A2** and **B1-B2)** Acetylated tubulin staining of 13 hpf zebrafish larvae showing presence of cilia (white) in the head region in both control **(A1)** n=12 and *elipsa* mutant **(B1)** n=5**. (A2, B2)** Insets drawn in the region near the neural tube to indicate presence of cilia in control **(A2)** and mutant **(B2)**. **(A3, B3)** Kupffer's vesicle labeling with acetylated tubulin (white) show similar numbers of cilia at 13hpf in control **(A3)** n=12 and mutant **(B3)** n=5**.**

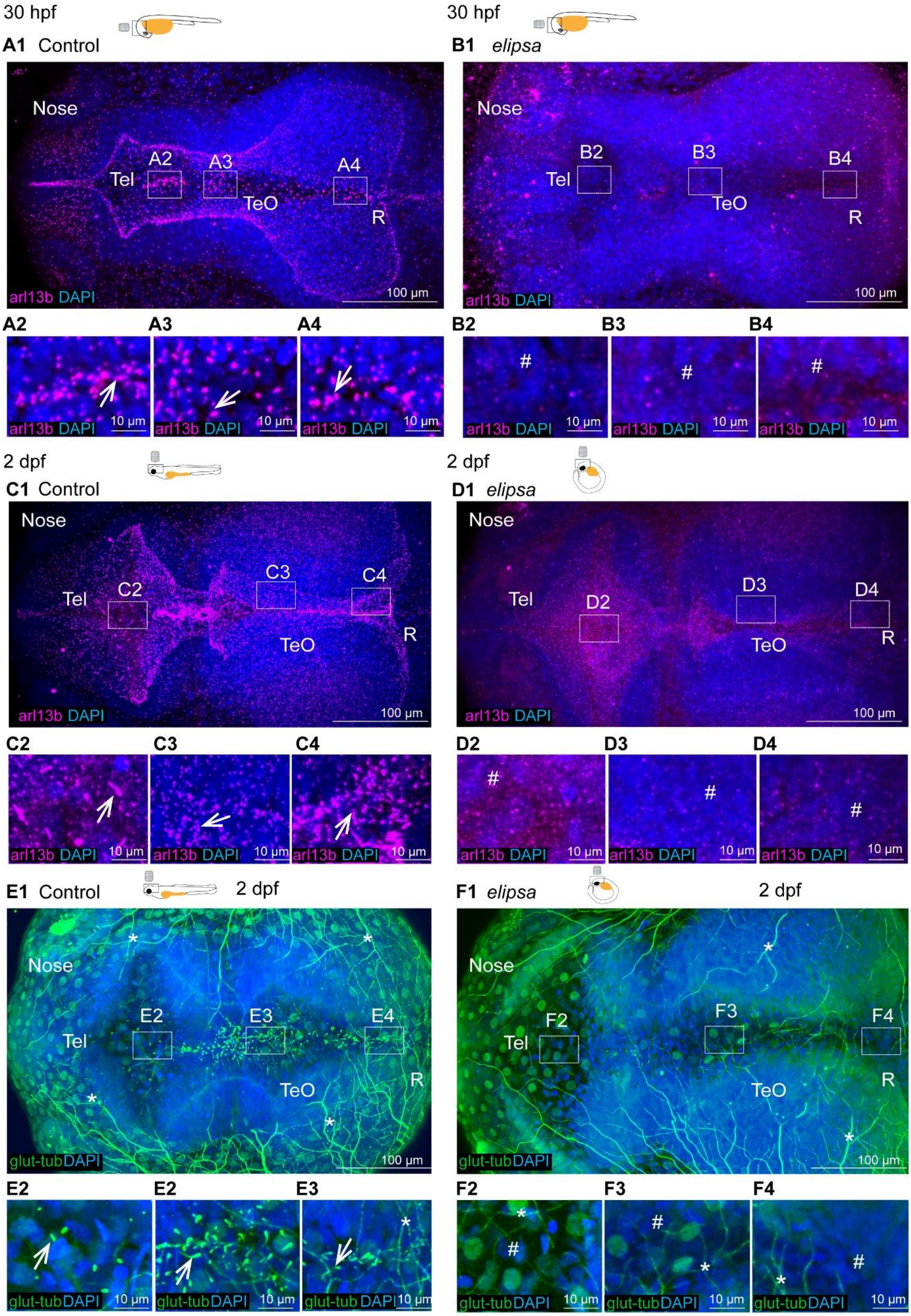
30 hpf
A1 Control
Nose
Tel
A2
A3
A4
TeO
R
arl13b DAPI
100 µm
30 hpf
B1 elipsa
Nose
Tel
B2
B3
B4
TeO
R
arl13b DAPI
100 µm
A2
A3
A4
arl13b DAPI
10 µm
B2
B3
B4
#
arl13b DAPI
10 µm
2 dpf
C1 Control
Nose
Tel
C2
C3
C4
TeO
R
arl13b DAPI
100 µm
2 dpf
D1 elipsa
Nose
Tel
D2
D3
D4
TeO
R
arl13b DAPI
100 µm
C2
C3
C4
arl13b DAPI
10 µm
D2
D3
D4
#
arl13b DAPI
10 µm
E1 Control
2 dpf
Nose
Tel
E2
E3
E4
TeO
R
*
glut-tub DAPI
100 µm
F1 elipsa
2 dpf
Nose
Tel
F2
F3
F4
TeO
R
*
glut-tub DAPI
100 µm
E2
E2
E3
glut-tub DAPI
10 µm
F2
F3
F4
#
*
glut-tub DAPI
10 µm

Supplemental figure S2: Cilia defects in the brain of 30hpf-2dpf *elipsa* mutant embryos. Related to figure 1.

(A1-A4 and **B1-B4)** Staining of 30hpf zebrafish larvae with arl3b antibody to stain all cilia in the brain, n=9 controls and 4 mutants. **A1** At 30 hpf, arl13b stained cilia are located in various brain regions, represented by insets drawn in different brain regions **(A2-A4). (B1)** Loss of cilia in the entire brain of *elipsa*, represented by insets drawn in different regions **(B2-B4).** (**C1-C4** and **D1-D4**) Staining of 2dpf brains with arl13b antibody to stain cilia in the brain, n=3 controls and 3 mutants. **(D1)** Loss of cilia in *elipsa* mutant brains, as shown by insets drawn in different regions **(D2-D4).** (**E1-E4** and **F1-F4**) Glutamylated tubulin staining for motile cilia at 2dpf, n=3 controls and 4 mutants. **(E1) S**ingle glutamylated tubulin-positive cilia are located in the forebrain on the dorsal roof and ventral part of the tectal/diencephalic ventricle and in the rhombencephalon, as indicated by insets drawn in the telencephalon **(E2)**, optic tectum**(E3)** and rhombencephalon **(E4). (F1)** *elipsa* mutants show a total loss of glutamylated tubulin-positive cilia in the brain, represented by insets drawn in different regions **(F2-F4).** Tel, Telencephalon; Teo, Optic Tectum; R, Rhombencephalon. Cilia loss is indicated by # and nonspecific signal from glutamylated tubulin antibody by *.

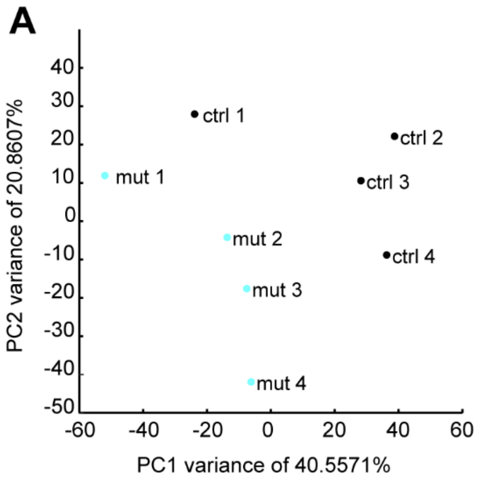

Supplemental figure S3: Principal Component Analysis (PCA) of RNA sequencing data. Related to figure 4

Examination of sample variation of RNA sequencing samples. Each dot represents a single biological replicate indicated in black for control and cyan for *elipsa* mutant. The number indicate the batch number.

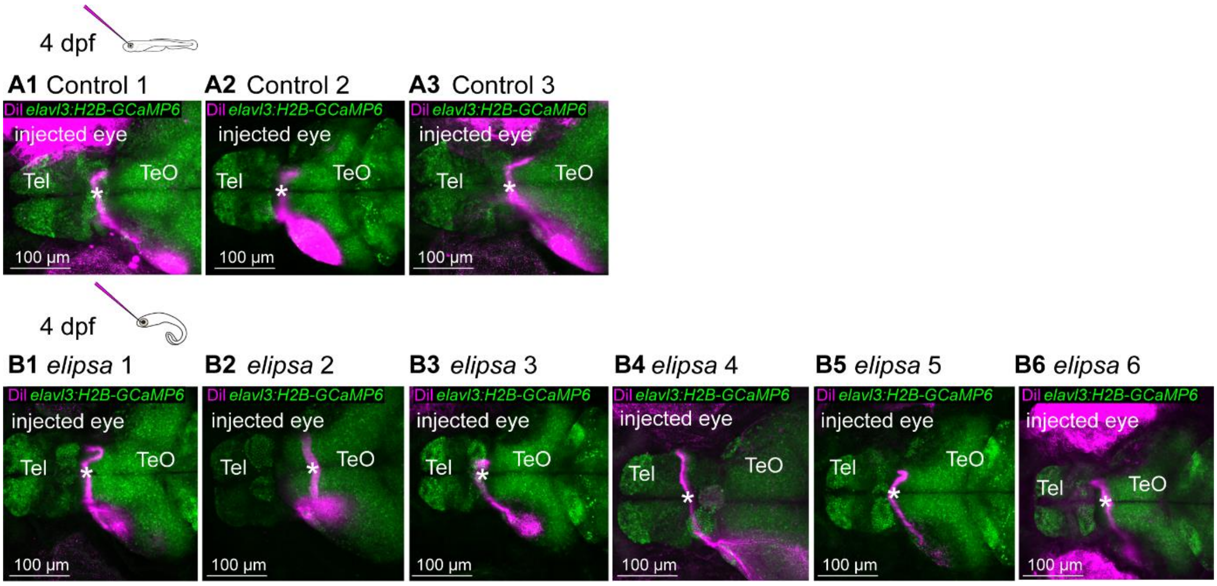

Supplemental figure S4: Abnormal axonal projections from the retinal ganglion cells to the tectum in control and *elipsa* mutant. Related to figure 5.

Examples of DiI injection into the eye at 4dpf to label the axonal projections of retinal ganglian cells in control **(A1-A3)** and elipsa **(B1-B4)**. * indicates region of optic chiasm.

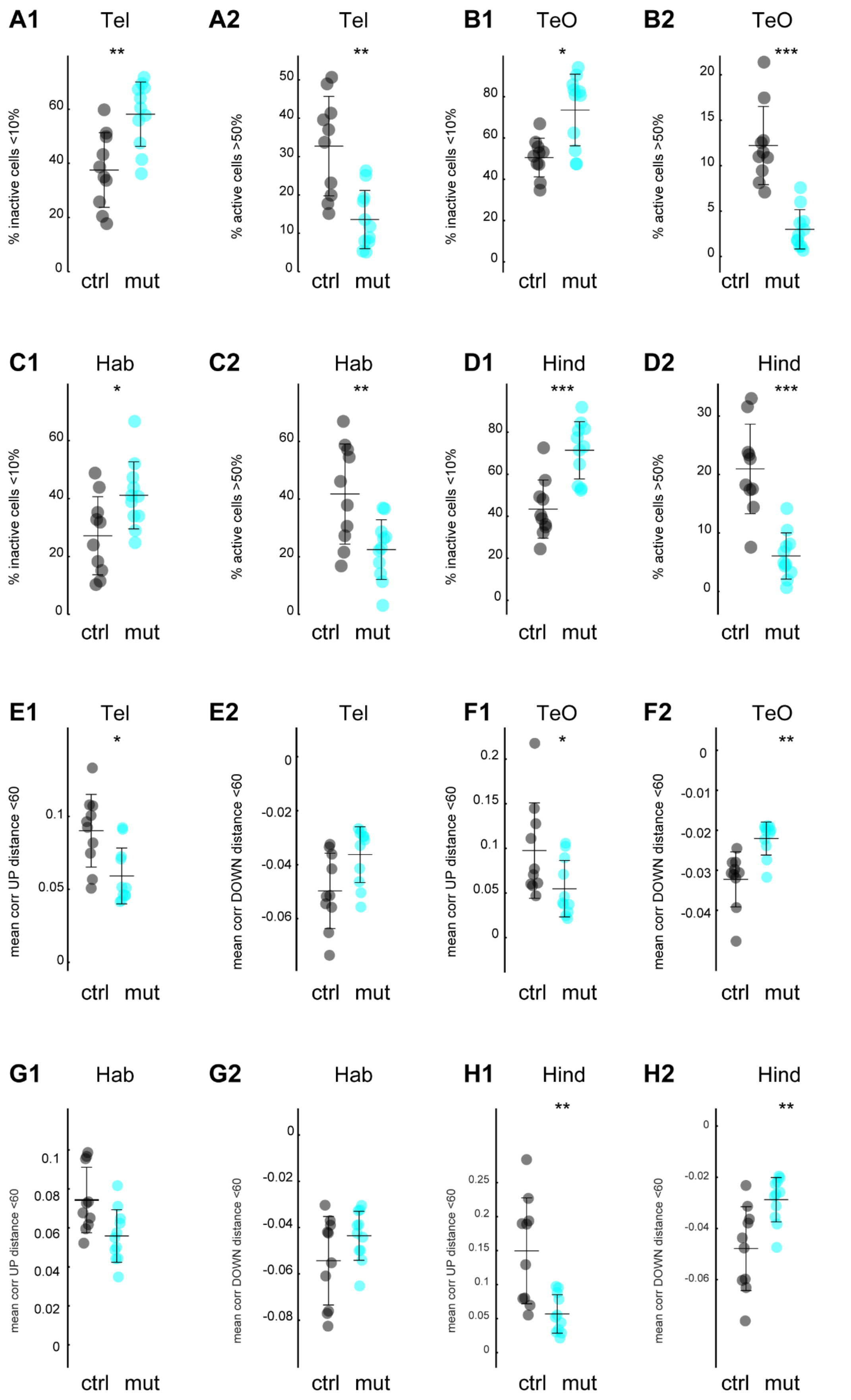

Supplemental figure S5: Reduced spontaneous activity and correlation between neighboring neurons in the brain of cilia mutants. Related to figure 7

(A1-D2) Quantification of inactive and active cells for the **(A1-A2)** telencephalon, **(B1-B2)** optic tectum and **(C1-C2)** habenula and **(D1-D2)** hindbrain regions, controls in black and *elipsa* mutant in cyan. N=10 controls and 11 mutants. **(E1-H2)** Quantification of mean positive (UP) and negative (DOWN) correlation for cells located within 60 µm distance in the **(E1-E2)** telencephalon, **(F1-F2)** optic tectum, **(G1-G2)** habenula and **(H1-H2)** hindbrain regions, controls in black and *elipsa* mutant in cyan. N=10 controls and 11 mutants. Statistical significance by Wilcoxon Rank Sum test, *: $p<0.05$, **:$p<0.01$, ***: $p < 0.001$. Tel, Telencephalon; Teo, Optic Tectum; Hab, Habenula; Hind, Hindbrain.
